# Assessing the impact, genomics and evolution of type II secretion across a large, medically important genus: the *Legionella* type II secretion paradigm

**DOI:** 10.1099/mgen.0.000273

**Published:** 2019-06-05

**Authors:** Richard C. White, Nicholas P. Cianciotto

**Affiliations:** 1 Department of Microbiology and Immunology, Northwestern University Medical School, Chicago, IL 60611, USA; ^†^​ Present address: Department of Genomic Medicine, J. Craig Venter Institute 9605 Medical Center Dr., Rockville, MD 20850, USA.

**Keywords:** *Legionella*, type II secretion, T2SS, Legionnaires' disease, *Legionella pneumophila*, *Aquicella*

## Abstract

The type II secretion system (T2SS) plays a major role in promoting bacterial survival in the environment and in human hosts. One of the best characterized T2SS is that of *Legionella pneumophila*, the agent of Legionnaires’ disease. Secreting at least 25 proteins, including degradative enzymes, eukaryotic-like proteins and novel effectors, this T2SS contributes to the ability of *L. pneumophila* to grow at low temperatures, infect amoebal and macrophage hosts, damage lung tissue, evade the immune system, and undergo sliding motility. The genes encoding the T2SS are conserved across the genus *Legionella*, which includes 62 species and >30 pathogens in addition to *L. pneumophila*. The vast majority of effectors associated with *L. pneumophila* are shared by a large number of *Legionella* species, hinting at a critical role for them in the ecology of *Legionella* as a whole. However, no other species has the same repertoire as *L. pneumophila*, with, as a general rule, phylogenetically more closely related species sharing similar sets of effectors. T2SS effectors that are involved in infection of a eukaryotic host(s) are more prevalent throughout *Legionella*, indicating that they are under stronger selective pressure. The *Legionella* T2SS apparatus is closest to that of *Aquicella* (another parasite of amoebae), and a significant number of *L. pneumophila* effectors have their closest homologues in *Aquicella*. Thus, the T2SS of *L. pneumophila* probably originated within the order *Legionellales*, with some of its effectors having arisen within that *Aquicella*-like progenitor, while other effectors derived from the amoebal host, mimiviruses, fungi and less closely related bacteria.

**Impact Statement**

In Gram-negative bacteria, the type II secretion system is notable for its wide-reaching impact on bacterial physiology, ecology and pathogenesis. This is especially true for *Legionella pneumophila*, the agent of Legionnaires’ disease. While giving an update on all aspects of type II secretion, this review provides a genomic assessment of the secretion system across the genus *Legionella* as well as hypotheses on how its evolution has been driven by bacterial interactions with amoebal host cells and other environmental microbes.

## *Legionella* taxonomy and pathogenesis

The genus *Legionella* was first recognized in the late 1970s, with the characterization of *Legionella pneumophila* as the aetiological agent of a form of pneumonia now known as Legionnaires’ disease [1, 2]. Within the *G* *amma* *proteobacteria*, *Legionella* is the sole genus contained within the family *Legionellaceae* [3]. Members of this genus are Gram-negative bacteria found ubiquitously in the environment in both freshwater systems such as lakes and rivers, as well as man-made aquatic systems [4–7]. There are at least 63 confirmed species of *Legionella* [8–14]. Additionally, there are a plethora of uncultured *Legionella-*like organisms in freshwater systems that may represent novel species [15–18]. Of the confirmed *Legionella* species, which fall into three major phylogenetic clades, 32 are disease-causing, based on cultures obtained from symptomatic individuals or seroconversion. However, approximately 90 % of cases of Legionnaires’ disease in the USA and Europe are caused by *L. pneumophila* [19]. Within aquatic systems, *L. pneumophila* and other legionellae primarily parasitize free-living protozoa. The host range of *Legionella* species is exceptionally broad, as co-isolation and co-culture experiments implicate permissive hosts within seven of the eight phyla within the protozoan kingdom, 12 of 41 classes within those phyla, and 21 of 82 known orders [20]. Some of the most abundant protozoa in nature, including species of *Acanthamoeba*, *Naegleria* and *Vermamoeba*, permit *L. pneumophila* replication and have been isolated from *Legionella-*containing waters [21–25]. Based on the results of assays done in the laboratory, *L. pneumophila* replicates and/or survives within at least 11 other genera of protozoa, including *Balamuthia*, *Ciliophrya*, *Dictyostelium*, *Echinamoeba*, *Hartmannella*, *Oxytricha*, *Paramecium*, *Stylonychia*, *Tetrahymena*, *Tetramitus* (formerly *Vahlkampfia*) and *Willaertia* [14, 20]. During human infection, *L. pneumophila* primarily grows within resident alveolar macrophages in the infected lung [26]; however, intracellular infection of type I and II alveolar epithelial cells may also contribute to the pathogenesis of Legionnaires’ disease [27, 28]. Within phagocytes, whether amoebae or macrophages, *L. pneumophila* evades fusion with lysosomes and instead modulates endoplasmic reticulum (ER)-to-Golgi vesicular trafficking to remodel the nascent phagosome into an ER-derived compartment known as the *Legionella*-containing vacuole [29]. For its intracellular lifestyle, *L. pneumophila* employs a type IV secretion system (T4SS), the Dot/Icm type IVB system, to deliver >300 proteins (effectors) into the cytosol of infected cells and directly target host processes including autophagy, death pathways, protein translation and turnover, as well as innate immunity [30]. *L. pneumophila* encodes a second T4SS, the Lvh type IVA system, which is similar to the Vir T4SS of *Agrobacterium tumefaciens* identified [31]. Although the VirD4 coupling protein within the Lvh apparatus has been implicated in bacterial entry into host cells and the subsequent evasion of phagosome acidification, no secreted effectors have yet been identified [32]. *L. pneumophila* also has a functional type I secretion system; however, this system is not required for intracellular growth, although it does enhance bacterial entry into host cells via its secretion of an RtxA-like toxin [33]. The bacterium also secretes a siderophore (rhizoferrin) and a melanin-like pigment, both of which promote iron acquisition and, in the case of rhizoferrin *L. pneumophila* growth in the lungs [34–37]. However, another major facet of the natural history and pathogenesis of *L. pneumophila* is the Lsp type II secretion system (T2SS) [38–42]. Combining experimental data obtained from studies done on *L. pneumophila* with the recent explosion in the genomic database, this review provides an up-to-date assessment of the impact of T2SSs across the genus *Legionella*, with added attention given to the variations in output of the Lsp system as well as thoughts on the evolution of this important secretion system. Since the *L. pneumophila* system represents one of the most well-characterized T2SSs [43–45], the topics and concepts covered in this review may be helpful for the evaluation of T2SS in other bacterial genera.

## General overview of the bacterial T2SS

### Mechanism of protein secretion by the T2SS

**Fig. 1.**
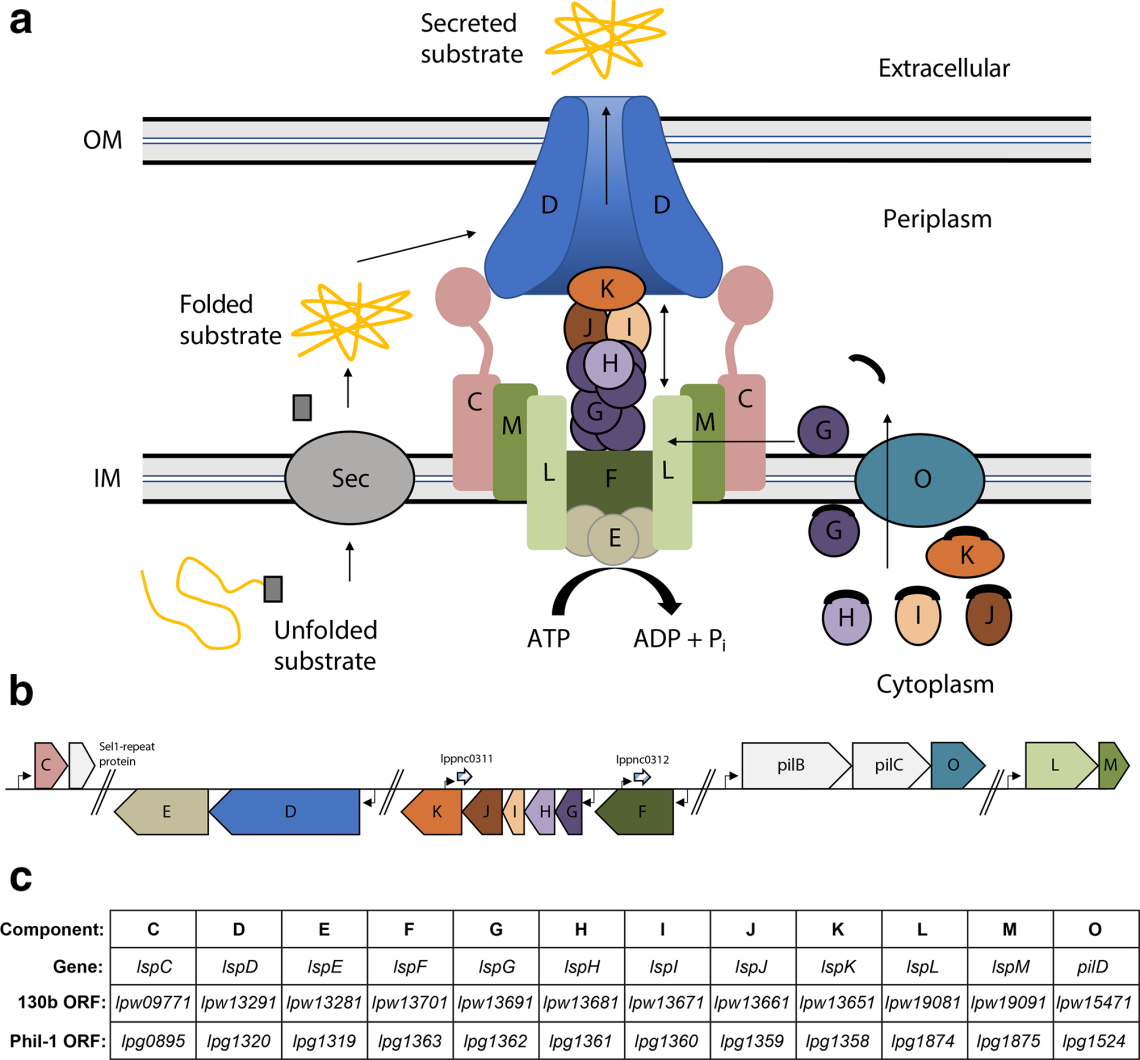
Overview of *L. pneumophila* T2SS. (a) Proteins containing a secretion signal peptide are first translocated across the inner membrane (IM) by the general secretory pathway (Sec) or the twin-arginine translocation pathway (Tat) (not shown). In the periplasm, the signal peptide is cleaved off, and the protein is folded into its tertiary form, and finally secreted into the extracellular milieu by the T2SS apparatus. The T2SS apparatus consists of inner transmembrane proteins (T2S F, L, M), which provide a platform for T2S E to bind. T2S E is a cytoplasmic ATPase which generates energy required to push proteins through the outer membrane (OM) secretin pore (T2S D). T2S O processes the major (T2S G) and minor (T2S H, I, J, K) pseudopilins before they are integrated into the T2SS apparatus, forming a pilus-like structure. T2S C links the inner and outer membrane components and facilitates substrate recognition in the periplasm. (b) Schematic of the five genomic loci encoding Lsp proteins. The distinct loci are separated by double slashes. The individual T2SS genes are indicated by the unique letter associated with the corresponding protein (e.g. D refers to the gene encoding the LspD/T2S D protein) and are coloured to match the colour of the corresponding protein in (a). Promoters are indicated by the black L-shaped arrows. Non-coding RNAs (ncRNA) are indicated by small hatched arrows with the lppnc designation corresponding to the ncRNA found in *L. pneumophila* strain Paris [73]. Linked genes that do not encode components of the T2SS appear in light grey. All gene arrows are drawn to scale. (c) Overview of gene names and ORF designations for the various T2SS components of *L. pneumophila*. 130b: *L. pneumophila* strain 130b; Phil-1: *L. pneumophila* strain Philadelphia-1.

First described in *Klebsiella oxytoca* [46, 47], type II secretion (T2S) is a two-step process for secreting proteins into the extracellular space. During T2S, unfolded protein substrates containing a signal sequence are first translocated across the bacterial inner membrane via the Sec pathway (Fig. 1a) [48, 49]. In the periplasm, the proteins are folded into their tertiary conformation, and are destined for translocation across the outer membrane via a multiprotein apparatus, the T2SS [50, 51]. In some instances, nascent proteins that fold within the cytoplasm and are moved across the inner membrane via the twin-arginine translocon (Tat) can be recognized by and secreted via the T2SS apparatus [49]. In *L. pneumophila* and a variety of other Gram-negative bacteria [43, 52], the T2SS is composed of 12 ‘core’ components that are required for biogenesis of the apparatus and secretion of substrates (Fig. 1a). Four inner membrane proteins (T2S C, T2S F, T2S L, T2S M) form an assembly platform (AP) to which a cytoplasmic ATPase (T2S E) binds [52–57]. After being processed by an inner membrane peptidase (T2S O), a major pseudopilin (T2S G) and four minor pseudopilins (T2S H, T2S I, T2S J, T2S K) assemble into an envelope-spanning pilus-like structure [58–61]. The T2S G protein interacts with T2S L, and this interaction is thought to promote pseudopilus biogenesis [62]. Powered by the T2S E ATPase, the pseudopilus appears to act as piston or an Archimedes screw to push folded substrates through a homomultimeric secretin pore (T2S D) in the outer membrane and thereby complete the secretion of the substrates into the extracellular milieu [48, 50, 52, 63] (Fig. 1a). The T2S C protein links the AP and outer membrane components [44, 64, 65] and appears to have a substantial role in substrate recognition [44, 66–70]. Compatible with secretion occurring in a species-specific manner, T2S C is among the least conserved proteins amongst the various T2SSs; for example, in *Vibrio* and *Dickeya* species, the protein possesses a PDZ domain, whereas in *Pseudomonas* spcies, it has a coiled-coil domain, and in *L. pneumophila*, a shorter T2S C has no known domain at its C terminus [44, 67, 69, 71, 72]. Biochemical and structural studies further suggest that T2SS effectors may also directly interact with T2S L and T2S M in the AP, as well as with the minor pseudopilins and the secretin T2S D [70]. The signal contained within the substrates themselves that is recognized by the T2SS remains poorly defined, although proteins secreted by T2S are often rich in β-strands [64].

In some Gram-negative bacteria, a lipidated protein called PulS/OutS or the ‘pilotin’ is required for the proper transport and targeting of the T2S D secretin to the outer membrane [74]. However, a canonical pilotin has not been found to be encoded within the *L. pneumophila* genome, based upon the use of a hidden Markov model (HMM) [75] to search for homologues of PFAM ID PF09691 [44, 76]. An alternative pilotin, AspS, has been described for *Vibrio*-type T2SSs [77], although an HMM search using PFAM ID PF16549 also failed to return any significant hits within the *L. pneumophila* genome. The apparent absence of a pilotin could suggest that the *Legionella* T2S D secretin (also known as LspD, see below) is capable of directing itself to the outer membrane, as has been proposed for the secretins of *Pseudomonas* and *Xanthomonas* T2SSs [78]. While the *Pseudomonas aeruginosa* HxcQ and *Xanthomonas campestris* XpsD possess both a Type II/lipoprotein signal peptide and an N-terminal lipobox motif [78], the DOLOP server [79] suggests that the *Legionella* secretin lacks these lipoprotein features; thus, transport of the *Legionella* secretin to the membrane may be accomplished via a novel mechanism. The *Legionella* secretin does, however, possess a predicted peptidoglycan-binding SPOR domain at the N terminus, which may facilitate its localization to the cell membrane, as does the peptidoglycan-binding PilQ secretin of *P. aeruginosa* [80, 81]. *L. pneumophila* also does not possess equivalents of T2S N (sometimes generally referred to as GspN), T2S A (GspA) or T2S B (GspB), proteins that are variably present across the T2SSs and are generally dispensable for secretion function [82–84]. Overall, the bacterial T2SS is evolutionarily related to type IV pili (T4P) [85], and the T2S O protein is required for both T2S and T4P biogenesis in *L. pneumophila* and others [40].

### Genome organization of the *Legionella* T2SS

Initially based on the sequencing of the clinical isolates Philadelphia-1, Paris, Lens, Corby, Alcoy and 130b (also known as strain Wadsworth or AA100) [86–90], the genes encoding the *L. pneumophila* T2SS are present within five distinct chromosomal loci (Fig. 1b, c). This is in contrast to the T2SS of most other organisms that possess T2SS genes encoded within a single operon [91–95]. Promoter analysis and transcriptional-start-site mapping in *L. pneumophila* strain Paris [73] confirmed that *lspF* is monocistronic, whereas the other *lsp* genes are co-transcribed with other genes (Fig. 1b). The *lspC* gene is the first gene in a two-gene operon, with the second gene encoding a Sel-1 repeat-containing protein that possesses a secretion signal peptide. The *lspO* (*pilD*) gene is co-transcribed with the T4P-associated genes *pilB* and *pilC*, as in *Aeromonas hydrophila* and others [96]. Strand-specific total RNA sequencing of strain Paris also revealed *cis*-encoded, anti-sense RNAs within the *lspFGHIJK* gene cluster [73].

### T2SS in other Gram-negative bacteria

**Fig. 2.**
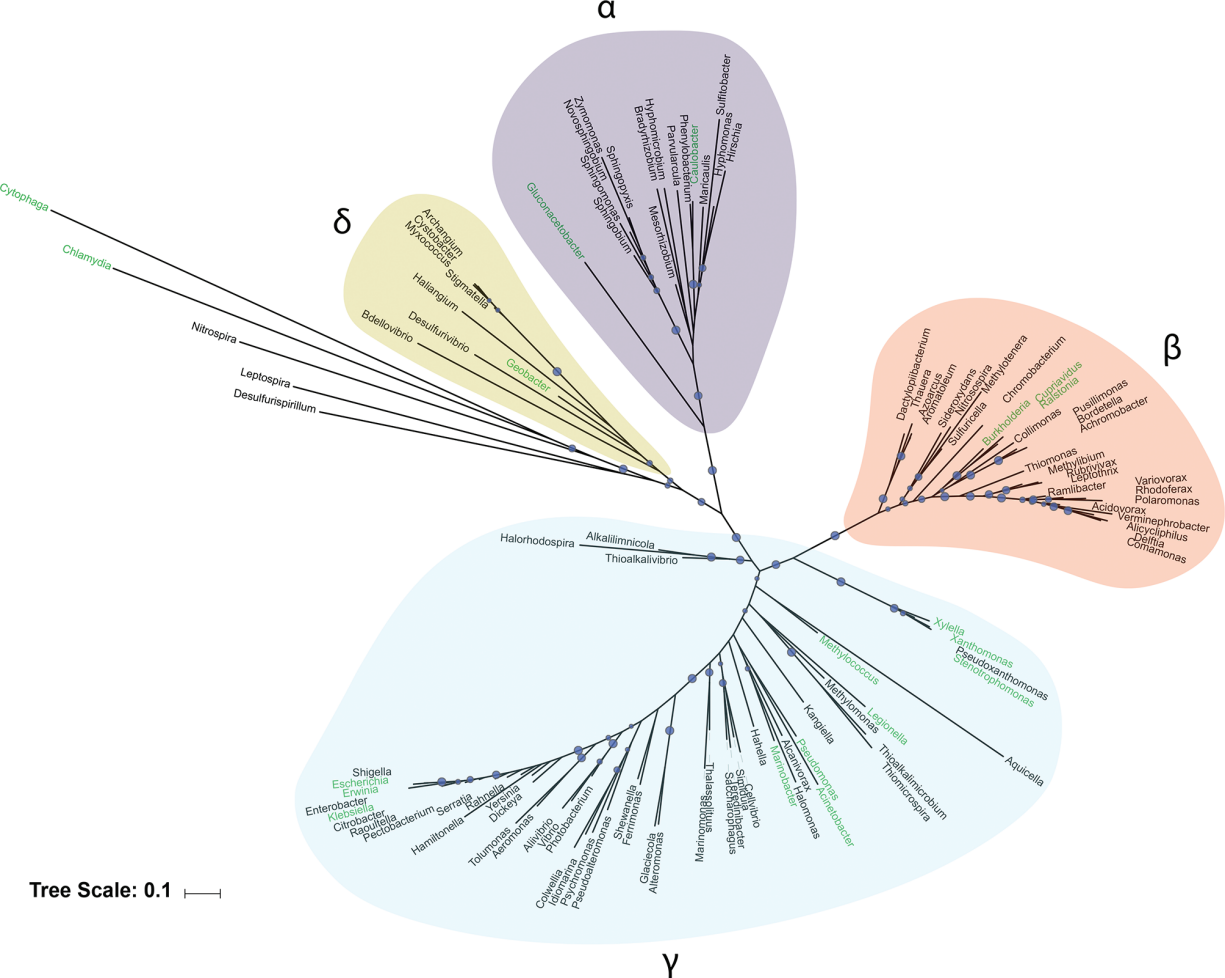
Distribution of T2SS genes among the *Proteobacteria* and beyond. An unrooted maximum-likelihood phylogenetic tree of *Proteobacteria* and other bacteria encoding a complete or near-complete T2SS was constructed using aligned 16S rRNA gene sequences [110] in RaxML (100 bootstrap replicates, GTR+Γ model) [111]. Bootstrap support values >50 are presented directly on the branches as grey circles, with larger circles corresponding to higher support values. Bar, 0.1 nucleotide substitutions per site. Clades are colour-coded by class of *P* *roteobacteria*, identified by the respective Greek symbols. Bacterial genera that encode a functional T2SS are coloured in green. Genera that are predicted to encode a T2SS but without demonstrated functionality are coloured in black.

Many T2SSs have now been characterized, mostly within numerous (but not all) genera in the *A* *lpha*-, *B* *eta*-, *G* *amma*- and *D* *elta* *proteobacteria*, although they have also been detected outside of the *P* *roteobacteria* [43, 50] (Fig. 2). Genome analysis of various *E* *psilon* *proteobacteria* spanning 15 genera suggests the T2SS is absent from this class of *P* *roteobacteria* (Table S1, available in the online version of this article). Some phyla may possess T2SSs that deviate from the canonical T2SS found among *P* *roteobacteria*; for example, secretion has been linked to T2SS-like genes in *Chlamydia trachomatis* and *Cytophaga hutchisonii* yet their genomes lack a complete set of T2SS genes [43, 97, 98]. Some bacteria, including strains of *Escherichia coli*, *Pseudomonas aeruginosa* *,* *Stenotrophomonas maltophilia* and *Yersinia enterocolitica*, possess two or more distinct T2SSs [43, 99]. The number of proteins secreted via T2S varies among the different bacteria, ranging from one in *K. oxytoca* to >60 in *L. pneumophila* and *Acinetobacter nosocomialis* [43] (see below). The substrates of T2S are generally delivered into the extracellular milieu; however, in a minority of cases, they can associate with the bacterial cell surface [45]. T2SSs often tend to secrete degradative enzymes such as proteases and peptidases, lipases, and carbohydrate-degrading enzymes that presumably aid in nutrition acquisition, among other things [43]. In addition to promoting the survival of numerous environmental bacteria, T2SSs can enhance the virulence attributes of animal pathogens (e.g. *Acinetobacter baumannii*, *Aeromonas hydrophila*, *Burkholderia pseudomallei, Chlamydia trachomatis, E. coli*, *Klebsiella pneumoniae*, *L. pneumophila*, *Photobacterium damselae*, *P. aeruginosa, S. maltophilia*, *V. cholerae, V. vulnificus* and *Y. enterocolitica*) and plant pathogens (e.g. species of *Dickeya, Erwinia*, *Pectobacterium, Ralstonia, Xanthomonas* and *Xylella*) [43, 50, 100–109].

## Role of the *L. pneumophila* T2SS in the environment and in disease

Many studies have assessed the role of T2S in *L. pneumophila* fitness and growth in both the environment and within eukaryotic host cells [43]. Most of these studies were conducted by comparing the phenotype of the clinical isolate strain 130b to that of a mutant specifically lacking a component of the T2SS, such as the T2S D secretin (LspD), T2S E ATPase (LspE) or the T2S F inner membrane platform protein (LspF) [42]. Importantly, all 130b mutant phenotypes were reversed when an intact copy of the T2S protein gene was re-introduced into the mutant, thereby confirming the role of the T2SS. While the T2SS mutants grow and survive similarly to wild type when inoculated onto solid media [e.g. buffered-yeast-extract (BYE) agar] or into liquid bacteriological media (e.g. BYE broth) at 30 and 37 °C, they are substantially impaired for growth in the amoebal hosts *Acanthamoeba castellanii*, *Vermamoeba vermiformis*, *Naegleria lovaniensis* and *Willaertia magna* at 35–37 °C [25, 38–40, 42, 112, 113]. The importance of T2S for infection of acanthamoebae has also been documented through the analysis of an *lsp* mutant of strain Philadelphia-1 [38]. The T2S mutant growth defect in the amoebal hosts is several orders of magnitude, and the numbers of mutant bacteria only increase ~1 log after 72 h of co-culture compared to the numbers of wild-type bacteria that increase 3–4 log over the same period. This intracellular growth defect becomes even more pronounced when the bacterial–amoebal co-cultures are incubated at 22–25 °C [114]. *L. pneumophila* T2S mutants display difficulty either growing on an agar medium at 25 °C and below or surviving planktonically in tap water at low temperature [114, 115]. Thus, T2S promotes the environmental persistence of *L. pneumophila* within intracellular niches and in the planktonic phase over a range of ambient temperatures. T2S may also support environmental persistence by contributing to long-lasting colonization of biofilms; for example, a mutant lacking the T2S-dependent substrate Lcl (see below) is impaired in biofilm formation on glass or polystyrene surfaces within static cultures [116, 117]. In support of this, a mutant lacking the T2S O pre-pilin peptidase is unable to persist within biofilms formed in a dynamic flow-cell system [118]. *L. pneumophila* T2S also promotes, albeit indirectly, the production of surfactant and thereby facilitates sliding motility on semi-solid agar [119–121]. It is quite likely that this function of T2S further facilitates the spread and survival of *L. pneumophila* within the environment.

Turning our attention to aspects of disease, *L. pneumophila* mutants lacking the T2SS display reduced replication (~10-fold) during intracellular infection of human macrophages, including the U937 and THP-1 cell lines and mononuclear cells obtained from human volunteers [42, 122, 123]. Similar results were obtained when the infection assays used a murine alveolar macrophage cell line (MH-S) or bone-marrow-derived macrophages obtained from A/J mice [124]. *L. pneumophila* mutants lacking T2S also exhibit reduced growth within human A549 type II epithelial cells and WI-26 VA4 type I epithelial cells as well as in the murine alveolar epithelial cell line TC-1 [122, 124]. Within both human and murine macrophages, T2S, although not needed for entry or evasion of the lysosome, is required for optimal Rab1B association with the *Legionella*-containing vacuole (LCV) and subsequent intravacuolar growth between 8 and 12 h post-infection [125]. Relatively early in the intracellular infection cycle, at least some of the T2S substrates translocate out of the LCV and reside nearby in the macrophage cytoplasm [126]. In the A/J mouse model of pneumonia, *L. pneumophila* T2S mutants are severely impaired in the ability to cause disease and showed no evidence of replication within the lungs [42]. Since the T2S mutant is only modestly impaired during *in vitro* infection of macrophages and epithelial cells, this observation suggests that the *L. pneumophila* T2SS promotes processes in addition to intracellular infection [124]. Compatible with such a scenario, the T2SS is also required for a dampening of the innate immune response of macrophages that is induced via the MyD88 and Toll-Like Receptor 2 signalling pathways [123, 124]. In support of these data concerning the various defects exhibited by T2SS mutants as compared to the parental strain, qRT-PCR analysis and other gene expression and transcriptome analyses have confirmed that the T2SS apparatus genes are significantly expressed by wild-type *L. pneumophila* during growth in bacteriological media and upon intracellular infection of both macrophages and multiple types of amoebae [25, 76, 113, 115, 125, 127–130].

## Secreted substrates (effectors) of the *L. pneumophila* T2SS

Bioinformatic analysis of the genome of strain Philadelphia-1 revealed at least 60 putative substrates of the *L. pneumophila* T2SS, i.e. proteins that contain a signal sequence and are predicted to have an extracellular localization [131]. Based on both a proteomic comparison of culture supernatants obtained from wild-type *L. pneumophila* strain 130b versus an *lspF* mutant and assessments of enzyme activities in wild-type versus mutant supernatants, 25 secreted proteins/activities of strain 130b were confirmed as being dependent upon T2SS (Table 1). In most cases, the proteins were later also detected in culture supernatants of other strains of *L. pneumophila* [132–134]. The vast majority of these confirmed T2SS substrates contain a typical signal sequence, indicating that they are moved across the inner membrane by the Sec translocon prior to incorporation into the T2SS [122]. The only exceptions are the phospholipase C PlcA and the putative peptidyl-proline *cis*/*trans*-isomerase (PPIase) LirB, which contain a twin-arginine motif and a twin-lysine motif, respectively, in their signal peptides and are translocated across the inner membrane via Tat rather than Sec [134, 135]. Interestingly, the secretion (or activation) of another phospholipase C activity, which is probably due to the PlcA-related PlcB [124], is dependent upon a surface-associated PPIase known as Mip [136]. In addition to being detected ‘free’ within culture supernatants, a number of the validated T2SS substrates are present within outer membrane vesicles (OMVs) (Table 1). As has been reported for other bacterial T2SSs, such a locale is a result of the substrates existing within the periplasm prior to completion of the secretion process as well as occurring, in some cases, on the bacterial cell surface [137–139]. An expanded proteomic analysis of supernatants obtained from cultures of *L. pneumophila* strain Philadelphia-1 and its derivative strain JR32 [132–134] has determined that another 47 putative substrates containing signal peptides are in fact secreted proteins (Table S2). Thus, the number of T2SS substrates produced by *L. pneumophila* is likely to be at least 72.

**Table 1.** Documented T2SS substrates of L. pneumophila*

T2SS substrate	Strain 130b ORF	Strain Phil-1 ORF	Protein activity or sequence novelty	Location(s)†	Role in infection ‡	Crystal structure	Prevalence within *Legionella*	Closest non-*Legionella* homologue	References
AmiA	*lpw03521*	*lpg0264*	putative amidase	Sup’t, OMV	may promote growth in A549, Ac, U937 and Vv		84 %	[*Bacteroidetes*] *Flagellimonas aquimarina* (56 % I, E=5×10^−70^)	[131, 133, 140]
CelA	*lpw19571*	*lpg1918*	endoglucanase	Sup’t, OMV	not required for growth in A549, Ac, Nl, U937, Vv, Wm and murine lung		39 %	[*Gammaproteobacteria*] *Methylococcus* spp. (24 % I, E=1×10^−10^)	[25, 113, 124, 131, 132, 141]
ChiA	*lpw11641*	*lpg1116*	chitinase	Sup’t, OMV	promotes growth in murine lung not required for growth in A549, Ac, Nl, U937, Vv and Wm		53 %	[*Gammaproteobacteria*] *Aquicella lusitana* (55 % I, E=1×10^−145^)	[25, 113, 124, 131, 132]
GamA	*lpw05041*	*lpg0422*	eukaryotic-like glucoamylase	Sup’t	not required for growth in Ac, Nl, U937, Vv and Wm		74 %	[Fungi] *Spizellomyces punctatus* (42 % I, E=8×10^−89^)	[25, 113, 142]
LapA	*lpw30701*	*lpg2814*	eukaryotic-like leu/tyr/phe/val/ile/met/asp aminopeptidase	Sup’t, OMV	promotes growth in Ac not required for growth in A549, Nl, U937, Vv, Wm and murine lung	PDB: 6ESL	95 %	[*Gammaproteobacteria*] *Aquicella lusitana* (43 % I, E=9×10^−109^)	[76, 113, 124, 131–133, 143]
LapB	*lpw00321*	*lpg0032*	eukaryotic-like lys/arg aminopeptidase	Sup’t	not required for growth in A549, Ac, Nl, U937, Vv, Wm and murine lung	PDB: 5GNE	16 %	[*Gammaproteobacteria*] *Aquicella lusitana* (40 % I, E=1×10^−89^)	[25, 76, 113, 124, 131, 143, 144]
Lcl	*lpw28961*	*lpg2644*	eukaryotic-like collagen-like protein	Sup’t, OMV, Surface	promotes attachment to A549, NCI-H292 and U937 promotes attachment and invasion of Ac and Vv not required for growth in Ac		11 %	[*Alphaproteobacteria*] *Sphingorhabdus flavimaris* (66 % I, E=3×10^−92^)	[116, 131, 132, 145, 146]
LegP	*lpw32851*	*lpg2999*	eukaryotic-like putative protease	Sup’t, OMV	not required for growth in Ac, Nl, U937, Vv and Wm		47 %	[*Alphaproteobacteria*] *Poseidonocella sedimentorum* (49 % I, E=3×10^−74^)	[113, 131, 132]
LipA	*lpw05481*	*lpg0468*	monoacylglycerol lipase	Sup’t	not required for growth in A549, Ac, Nl, U937, Vv, Wm and murine lung		89 %	[*Gammaproteobacteria*] *Berkiella cookevillensis* (40 % I, E=2×10^−66^)	[25, 113, 124, 131, 147]
LipB	*lpw12111*	*lpg1157*	triacylglycerol lipase	Sup’t	not required for growth in A549, Ac, Nl, U937, Vv, Wm and murine lung		54 %	[*Lentisphaerae*] *Victivallis vadensis* (36 % I, E=2×10^−40^)	[25, 113, 124, 131, 147]
LirB	*lpw20131*	*lpg1962*	putative peptidyl proline *cis-trans*-isomerase	Sup’t, OMV (Tat substrate)	not required for growth in Ac and HL-60		67 %	[*Nitrospirae*] *Leptospirillum ferriphilum* (67 % I, E=2×10^−74^)	[133, 134, 148, 149]
Map	*lpw11671*	*lpg1119*	eukaryotic-like tartrate-sensitive acid phosphatase	Sup’t, OMV	not required for growth in A549, Ac, Nl, U937, Vv, Wm and murine lung	PDB: 5CDH	49 %	[*Gammaproteobacteria*] *Francisella* spp. (39 % I, E=1×10^−81^)	[25, 113, 124, 131, 132, 150, 151]
NttA	*lpw13951*	*lpg1385*	novel	Sup’t	promotes growth in Ac and Wm not required for growth in Nl, U937 and Vv		77 %	None	[25, 113, 131]
NttB	*lpw28721*	*lpg2622*	Novel C1 family peptidase	Sup’t	not required for growth in Ac, Nl, U937, Vv and Wm	PDB: 6A0N	75 %	[*Gammaproteobacteria*] *Piscirickettsia salmonis* (35 % I, E=2×10^−48^)	[25, 113, 131, 152]
NttC	*lpw18401*	*lpg1809*	novel	Sup’t	promotes growth in Vv and Wm may promote growth in Ac not required for growth in Nl		86 %	None	[113, 131]
NttD	*lpw10421*	*lpg0956*	novel, DUF4785-containing protein	Sup’t	promotes growth in Ac not required for growth in Nl, U937 and Vv	PDB: 4KH9	84 %	[*Gammaproteobacteria*] *Dyella japonica* (25 % I, E=2×10^−22^)	[76, 131]
NttE	*lpw02811*	*lpg0189*	novel	Sup’t	may promote growth in Ac, Nl, U937 and Vv		65 %	None	[131, 153]
NttF	*lpw09571*	*lpg0873*	novel	Sup’t, OMV	may promote growth in Ac		91 %	[*Gammaproteobacteria*] *Piscirickettsia litoralis* (37 % I, E=5×10^−11^)	[131, 132, 153]
NttG	*lpw18641*	*lpg1832*	novel, VirK-like	Sup’t	not determined	PDB: 5XTA	58 %	[*Gammaproteobacteria*] *Francisella halioticida* (32 % I, E=6×10^−19^)	[131, 154]
PlaA	*lpw25361*	*lpg2343*	lysophospholipase A	Sup’t	promotes destabilization of the LCV not required for growth in A549, Ac, Nl, U937, Vv, Wm and murine lung		100 %	[Cyanobacteria] *Nostoc punctiforme* (34 % I, E=3×10^−47^)	[25, 113, 124, 131, 155, 156]
PlaC	*lpw30971*	*lpg2837*	glycerophospholipid: cholesterol transferase (GCAT), phospholipase A	Sup’t, OMV	promotes growth in Ac, Nl, Vv and Wm not required for growth in A549 and U937		77 %	[*Gammaproteobacteria*] *Parendozoicomonas haliclonae* (27 % I, E=7×10^−33^)	[25, 76, 113, 124, 132, 157]
PlcA	*lpw05821*	*lpg0502*	eukaryotic-like phospholipase C	Sup’t, OMV (Tat substrate)	not required for growth in A549, Ac, Nl, U937, Vv, Wm and murine lung		16 %	[*Gammaproteobacteria*] *Aquicella lusitana* (44 % I, E=3×10^−131^)	[25, 113, 124, 131, 132, 134, 147]
PlcB	*lpw14741*	*lpg1455*	eukaryotic-like phospholipase C	Sup’t, OMV	not required for growth in A549, Ac, Nl, U937, Vv and Wm		33 %	[*Gammaproteobacteria*] *Pseudomonas fluorescens* (38 % I, E=2×10^−63^)	[25, 113, 124, 132]
ProA	*lpw05471*	*lpg0467*	metalloprotease	Sup’t, OMV	promotes tissue destruction in lung promotes growth in Nl and Vv may promote growth in Ac not required for growth in A549, HL-60, U937, Wm, explanted guinea pig macrophages, and murine lung		100 %	[*Gammaproteobacteria*] *Aquicella lusitana* (43 % I, E=2×10^−138^)	[25, 113, 124, 131–133, 158–164]
SrnA	*lpw31111*	*lpg2848*	T2 ribonuclease	Sup’t	promotes growth in Nl and Vv not required for growth in A549, Ac, U937, Wm and murine lung		95 %	[*Gammaproteobacteria*] *Francisella philomiragia* (40 % I, E=1×10^−82^)	[25, 113, 124, 131, 158]

*Based on the presence of the indicated protein in wild-type culture supernatants and absence in T2SS mutant culture supernatants, plus the presence of a secretion signal at the N terminus of the predicted protein.

†Sup’t, protein is present in broth culture supernatant; OMV, also present in outer membrane vesicles; Surface, also present on the bacterial cell surface. Proteins that are predicted to be Tat, rather than Sec, substrates are indicated in parentheses.

‡Based on the behaviour of the corresponding mutant(s) in the indicated infection assay(s): ‘not required’, when the mutant was not different from wild-type; ‘promotes’, when the mutant was impaired relative to wild-type, and that defect was reversed by genetic complementation; ‘may promote’, when the mutant was impaired but genetic complementation has not yet been attempted or achieved. Ac, *Acanthamoeba castellanii*; Ap, *Acanthamoeba polyphaga*; Nl, *Naegleria lovaniensis*; Vv, *Vermamoeba vermiformis*; Wm, *Willaertia magna*; Dd, *Dictyostelium discoideum*

**Fig. 3.**
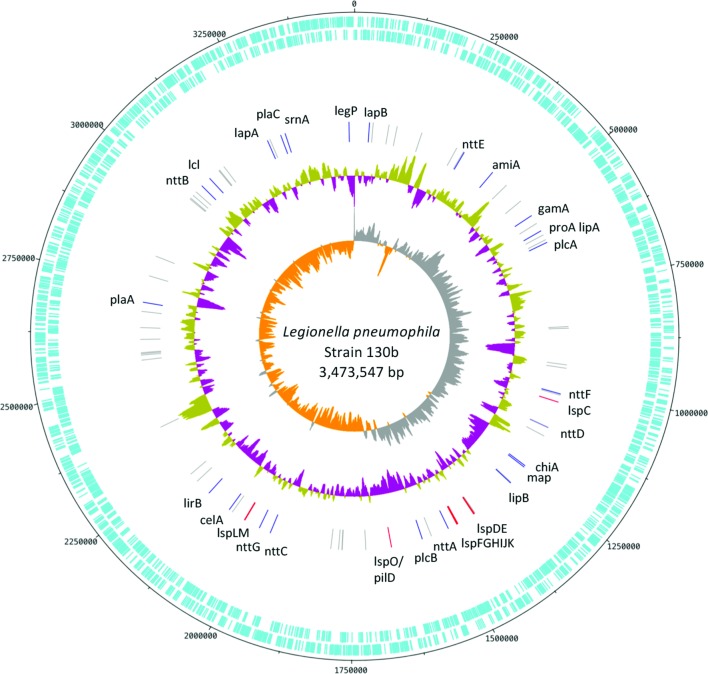
Chromosomal organization of T2SS genes in *L. pneumophila* strain 130b. The entire chromosome is depicted as a circular map. The tick marks indicate the nucleotide position from 0 to ~3 500 000 bp along the circular chromosome, with 0 indicating the origin of replication. From outside in, the two bands (in aqua) depict the predicted coding sequences transcribed clockwise and anticlockwise, respectively. The next band inward indicates the genomic positions of known individual T2S effectors (blue lines, with gene names nearby), putative T2S effectors (grey) and T2SS apparatus genes (red lines, with gene names nearby). Further inside is the GC content, with gold indicating above average and purple indicating below average GC content relative to the genomic average (38.2 mol%). The inner-most band represents the GC skew, indicative of the preference for G (grey) or C (orange) base pairs.

Although additional work is needed to confirm the T2SS-dependency of the 47 candidate effectors, the studies that were mainly done using strain 130b have characterized 25 *bona fide* substrates of *L. pneumophila* T2S (Table 1). The location of the genes encoding these known T2SS substrates is shown in Fig. 3. It is apparent that the T2SS effector genes are scattered around the strain 130b chromosome as opposed to being localized to one or a few loci or a genomic island. A similar conclusion can be made from analysing the genomes of strains Philadelphila-1, Paris and Lens.

### Degradative enzymes and enzyme activities

Early studies of *L. pneumophila* revealed an abundance of extracellular enzyme activities, including chymotrypsin-like activity [165], caseinase and gelatinase [166, 167], serum protein degrading protease [168], aminopeptidases [169], phosphatase, lipase, deoxyribonuclease, ribonuclease, cellulase as well as starch hydrolysis [170]. It was later appreciated that many of these activities are secreted via the T2SS in *L. pneumophila* strain 130b, based on the reductions in activity that were observed in both *lsp* mutant and effector mutant supernatants [38–40, 76, 143, 171, 172] (Table 1). More limited mutant analysis done with strains Corby and JR32 confirmed the T2S-dependency of some of these enzymes across *L. pneumophila* strains [38, 142, 173].

When mutants lacking individual exoenzymes were analysed in infection assays, the ProA protease, SrnA ribonuclease, PlaC acyltransferase and LapA aminopeptidase proved to be required for optimal infection of amoebae, and interestingly the relative importance of each of these effectors varied depending upon the type of amoeba infected (Table 1). It is surmised that the degradation of amoebal proteins, peptides, RNA and lipids by these T2SS effectors promote nutrient (e.g. amino acids, nucleotides, phosphate, fatty acids) acquisition for intracellular growth, although other scenarios, such as enzyme-mediated modifications to the LCV, are also possible [76, 158]. ProA is also notable for being required for the cleavage and activation of the T2SS effectors LapA, LapB, PlaA and PlaC [76, 174, 175]. Indeed, the defect that the *proA* mutant exhibits in *V. vermiformis* is linked to the role ProA has in PlaC activation [25]. However, the *proA* mutant’s defect in *N. lovaniensis* is independent of the protease’s activation of LapA, LapB, PlaA or PlaC, suggesting that ProA may activate additional T2SS effectors or in some cases directly target the host to promote bacterial replication. In the case of LapA, the crystal structure of the T2SS effector has been recently determined, providing insight into the broad specificity of this aminopeptidase, which is active against >10 substrates [76] (Table 1). Incidentally, other known T2S-dependent exoenzymes that have had their structures resolved include the LapB aminopeptidase [76, 144] and Map acid phosphatase [150, 151] (Table 1).

Thus far, no known T2SS substrate, whether an exoenzyme or not, has been documented as being required in *L. pneumophila* growth in macrophages, suggesting functional redundancy among the effectors and/or the existence of other T2SS substrates that are more critical for infection of mammalian cells [124, 176–178]. A limited analysis has also failed to uncover a T2SS substrate that is required for intracellular growth in epithelial cells [124]. Intriguingly, the ChiA chitinase is needed for full bacterial growth in the lungs of infected A/J mice [131]. Since a *chiA* mutant appears to be normal for growth in macrophages and epithelial cells, it is not clear how the chitinase promotes intrapulmonary growth. However, given that mammals do not possess chitin, the T2SS effector must be degrading a ‘chitin-like’ molecule in the lungs and/or encoding another type of activity. Although ChiA is the only T2SS effector that has been shown to be required for bacterial survival in the lungs, ProA probably also contributes to disease by mediating the destruction of lung tissue [159, 161–164, 179]. Additionally, ProA may aid in both iron assimilation by degrading host transferrin and immune evasion by degrading cytokines [124, 180].

For three reasons, we suggest that the *L. pneumophila* T2SS elaborates multiple other secreted enzymes. First, while many secreted activities are completely abolished upon mutation of the corresponding substrate gene, there is residual chitinase activity in a *chiA* mutant and residual aminopeptidase activity in a *lapA lapB* double mutant, suggesting the existence of additional secreted enzymes with overlapping functions [131, 143]. Compatible with these observations, *L. pneumophila* encodes two more putative aminopeptidases [Lpw05621 (Lpg0482) and Lpw12101 (Lpg1156)] and one additional putative chitinase [Lpw24031 (Lpg2217)] (Table S2). Second, there are enzyme activities present in wild-type, but not *lsp* mutant, supernatants that have not yet been linked to a known T2S substrate. These activities include tartrate-resistant acid phosphatase, diacylglycerol lipase, peptidoglycan hydrolase, xylanase and DNase [140, 147, 150, 170, 171]. In line with these results, *L. pneumophila* secretes another putative lipase [Lpw10431 (Lpg0957)] as well as a putative xylanase [Lpw07891 (Lpg0712)] (Table S2). The *lsp* mutants of *L. pneumophila* strain 130b are also impaired for surfactant production, sliding motility and poly-3-hydroxybutyrate metabolism, suggesting the existence of yet additional T2SS effectors that may have enzymatic activity [120]. Third, based upon proteomic analysis of strain 130b, there are documented T2SS substrates that have strong sequence similarity to known enzymes in other bacteria (Table 1). These include both the LirB protein, which is a putative PPIase that is highly expressed at low temperatures [149], and the AmiA protein, which is likely to be an amidase [131]. For AmiA, Phyre2 analysis [181] identified, with 100 % confidence, the AmpD amidase from *Citrobacter freundii* [182] as the top template to model the tertiary structure of ~82 % of the AmiA protein (31 % identity over 168 residues, from amino acids 33 to 200). In a similar vein, functional annotation of the secretome of strain Philadelphia-1 suggests that 20 % of the T2SS effectors are peptidases [183], which is compatible with *L. pneumophila* having a tendency to use amino acids as its primary food [184–186].

For 15 of the T2SS-dependent exoenzymes, including ChiA, PlaC, ProA and SrnA, the protein has its greatest homology to proteins/enzymes encoded by various genera of *G* *amma* *proteobacteria* (Table 1), which is not unexpected given the position of *Legionella* within the *G* *amma* *proteobacteria* (Fig. 2). Interestingly, however, PlaA is most similar to proteins encoded within the cyanobacteria, and AmiA, Lcl, LegP, LipB and LirB, have close bacterial homologues in the *A* *lpha* *proteobacteria* or elsewhere (Table 1). Arguably, most interestingly, some T2SS substrates are either most closely related to a eukaryotic enzyme(s), as in the case of GamA, or seemingly restricted to the genus *Legionella* as in the case of NttA, NttC and NttE (Table 1).

### Eukaryotic-like domains within T2SS substrates

**Fig. 4.**
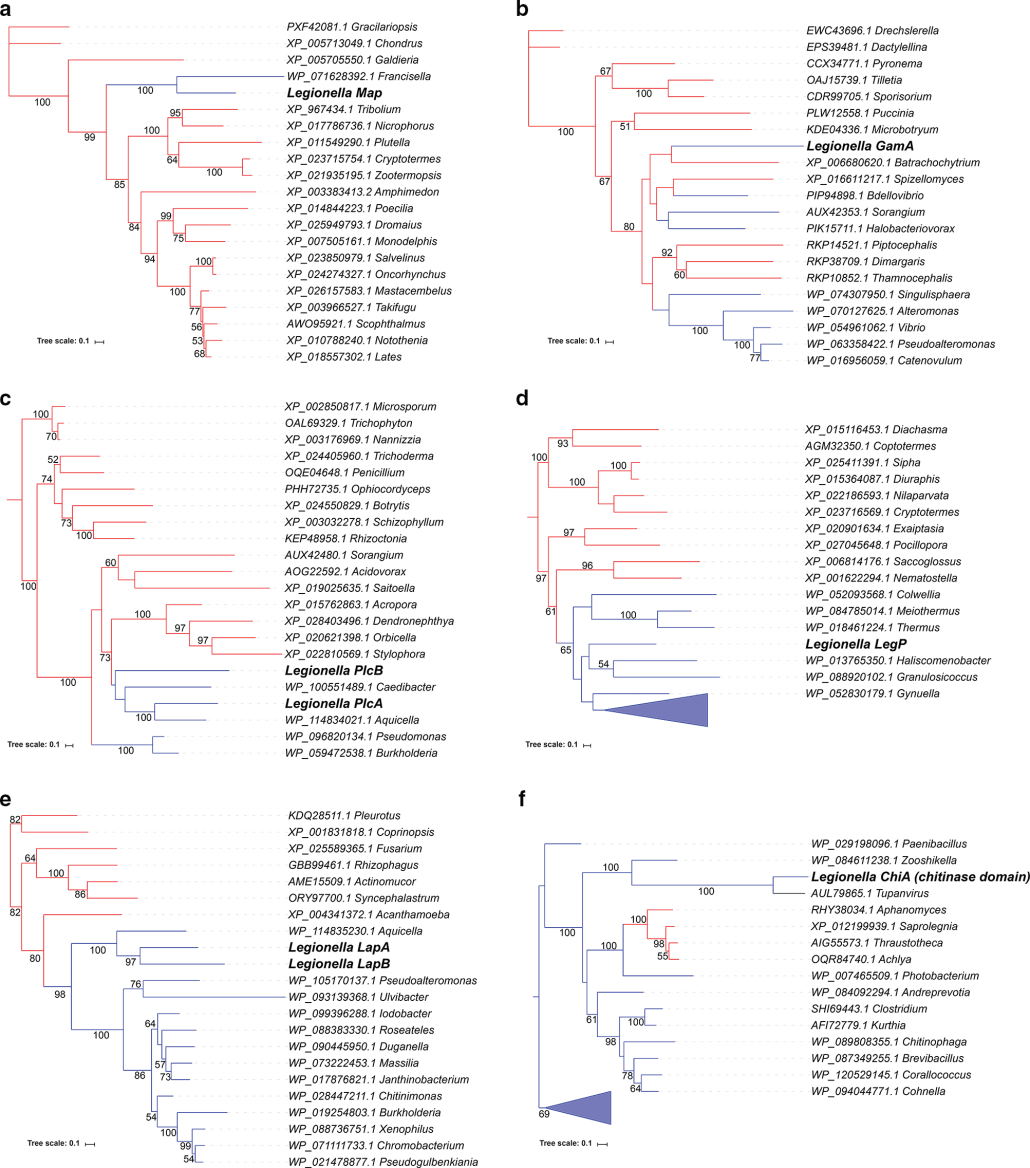
Phylogenetic analysis of select eukaryotic-like T2SS effectors of *L. pneumophila*. Homologues of *Legionella* T2SS effectors were identified by blastp using a minimum query coverage of 60 %, and amino acid identity and E-value cutoffs of 30 and 1×10^−30^, respectively, for panels a–e, and amino acid identity and E-value cutoffs of 25 and 1×10^−15^, respectively, for panel f. Maximum-likelihood trees were generated from full-length amino acid alignments of the T2SS effectors and the most closely related homologues encompassing at least 20 genera per effector group using RaxML (100 bootstrap replicates, GTR+Γ model) [111]. The trees of related sequences are given for the acid phosphatase Map (a), glucoamylase GamA (b), phospholipases PlcA and PlcB (c), putative astacin protease LegP (d), aminopeptidases LapA and LapB (e), and chitinase domain of ChiA (f). Bootstrap support values >50 are presented at the respective nodes. Bars, 0.1 amino acid substitutions per site. Monophyletic clades of bacterial homologues have been collapsed in panels d (*N*=70 genera) and f (*N*=16 genera) for space. Eukaryotes are indicated by red branches and bacteria by blue branches. GenBank accession numbers of the analysed protein sequences are listed before the respective genus designations.

Since the completion of the *L. pneumophila* genome, eukaryotic-like domains have been a hallmark of the effectors of the Dot/Icm T4SS, and it has been hypothesized that eukaryotic-like T4SS effectors were acquired via inter-kingdom horizontal gene transfer (HGT) [86, 187–190]. It is important to emphasize that eukaryotic-like domains also often exist among the known T2SS effectors. As reported in 2001, the first such-characterized substrate is the histidine acid phosphatase Map (150). Although Map is most closely related to a histidine-type phosphatase of *Francisella* spp. (Table 1), phylogenetic analysis reveals that the *Legionella* (and *Francisella*) protein is most closely related to eukaryotic acid phosphatases such as those found among genera of red algae, including *Gracilariopsis*, *Chondrus* and *Galdieria* (Fig. 4a). Interestingly, *Francisella* species, the only other bacteria possessing a protein that is highly similar to Map, are, like *Legionella* species, capable of replication within macrophages and protozoa [191–194]. Thus, the HGT of Map from a eukaryotic host(s) may have facilitated the acquisition of some aspect of the intracellular lifecycle of these bacteria, although no infection phenotype has been described thus far for a *map* mutant [25, 113, 150].

Originally identified as a eukaryotic-like protein based on the presence of an amylase domain [86, 187], GamA is involved in the breakdown of the eukaryotic storage molecule glycogen [142]. Although GamA-like proteins with signal sequences are found among *G* *amma*-and *D* *elta* *proteobacteria*, blastp analysis reveals that GamA is most closely related to a protein of the fungus *Spizellomyces punctatus* (Table 1). Phylogenetic analysis of the most closely related GamA homologues spanning 20 genera confirms that GamA is most closely related to eukaryotic proteins (Fig. 4b). Despite the relatedness of GamA to eukaryotic proteins, *gamA* mutants are not impaired for intracellular infection [25, 142], suggesting that the protein has a dispensable role in *L. pneumophila* growth within host cells.

blastp analysis reveals that PlcA is most closely related to a putative phospholipase in *Aquicella lusitana*, whereas PlcB is most akin to a putative phospholipase in *Pseudomonas fluorescens* (Table 1). PlcA- and PlcB-like proteins are also present in other intra-amoebal parasites (or endosymbionts) such as other *Aquicella* and *Pseudomonas* species as well as *Burkholderia* and *Caedimonas* species [195–197], . Interestingly, however, the phospholipase C proteins PlcA and PlcB, which are 36 % identical and 56 % similar to each other, belong within the phosphatidylcholine-hydrolysing group of eukaryotic phospholipases C that spans from the yeast *Saitoella* to the marine corals *Acropora*, *Orbicella* and *Stylophora* [197]. Indeed, phylogenetic analysis supports the view that both PlcA and PlcB are eukaryotic-like (Fig. 4c), although neither protein has been found thus far to be required for intracellular infection (Table 1). Interestingly, a triple mutant lacking PlcA, PlcB and a Dot/Icm T4SS-dependent PLC (PlcC) is impaired in a *Galleria mellonella* infection model [173].

Another known T2SS effector of *L. pneumophila* that can be considered eukaryotic-like is LegP (Table 1). LegP contains an astacin-like protease domain [131, 187] and phylogenetically LegP-like proteins, although found in many bacterial Gram-positive and Gram-negative genera, are highly similar to putative proteases from marine eukaryotes including the cnidarians *Nematostella*, *Pocillopora* and *Exaiptasia*, as well as the ocean-dwelling worm *Saccoglossus* (Fig. 4d). LegP is also unusual for being translocated out of the LCV in a T4SS-dependent manner, while being secreted into the bacterial culture supernatants via the T2SS [131, 198]. Such a dual-secretion phenomenon may also apply to several of the putative substrates (Table S2). The molecular basis for this differential secretion is unknown. However, it was recently documented that *Vibrio parahaemolyticus* can secrete the TDH exotoxin into the extracellular milieu via both T2SS and a type III secretion system [199], lending support to the existence of dual secretion mechanisms.

LapA and LapB, which are 45 % identical and 63 % similar, are aminopeptidases that provide critical nutrients to *L. pneumophila* during intracellular infection of protozoa [76, 143]. LapA and LapB share high sequence homology with a secreted aminopeptidase of *A. castellanii*, which, as noted above, is one of the major hosts for *L. pneumophila* [76]. Phylogenetic analysis affirmed that LapA may have been acquired from a protozoan host, with other amoebal-parasites such as *Aquicella*, *Burkholderia* and *Duganella* species also possessing LapA-like proteins (Fig. 4e). On the other hand, LapB represents a more recent gene duplication, with LapB undergoing faster adaptation and possessing enzymatic activities distinct from LapA [76]. That protozoa were probably the direct source of genetic material for *Legionella* has been previously proposed for many T4SS substrates as well as some non-secreted proteins [200, 201].

Yet another eukaryotic-like effector is ChiA. Although most closely related to a hypothetical protein in the gammaproteobacterium *A. lusitana* (Table 1), ChiA possesses a family-18 chitinase domain that is most akin to glycosyl hydrolase domains encoded by mimiviruses that infect the amoebae *A. castellanii* and *V. vermiformis* [202]. Amino acid residues 445–782 of ChiA have high relatedness (56 % identity, 73 % similarity, E-value=3×10^−146^) to a mimivirus isolated from the ocean depths, whereas residues 443–782 share high amino-acid sequence homology (53 % identity, 70 % similarity, E-value 3×10^−137^) with another mimivirus isolated from a high-alkalinity/high-salinity lake. Given that mimiviruses and *L. pneumophila* have co-evolved with the protozoan host, it is not surprising that mimiviruses have been proposed as a source for HGT in *Legionella* species [189, 203, 204]. From phylogenetic analysis, the chitinase domain of ChiA also appears related to putative chitinases found in water moulds (Fig. 4f). Since water moulds were previously implicated in HGT with mimiviruses [205], we posit that mimiviruses may have been a conduit for *L. pneumophila* acquisition of the chitinase domain from water moulds. Thus, like LapA, ChiA may be an example of a T2SS effector that evolved as a result of *L. pneumophila* growth within amoebae.

As just described, 8/25 (32 %) of the known T2SS effectors are eukaryotic-like. Although the known T2SS substrate Lcl has the Gly-aaX-aaY collagen helix motif found originally in eukaryotes [116, 117, 131, 145], it and other bacterial collagen-like proteins primarily share similarities with eukaryotic proteins at the structural level [206]. Furthermore, the amino acids at positions X and Y within the collagen helix of Lcl are rather distinct from those found in eukaryotes [206]. Consequently, we would not consider Lcl to be a ninth eukaryotic-like T2SS effector. However, if one examines the other 47 putative substrates of the T2SS (Table S2), there are four additional eukaryotic-like effectors [i.e. Lpw03931 (Lpg0301), Lpw10571 (Lpg0971), Lpw24081 (Lpg2222) and Lpw28361 (Lpg2588)]. This suggests that at least 17 % (i.e. 12/72) of the *L. pneumophila* T2SS substrates are eukaryotic-like in nature. Thus, eukaryotic-like effectors of *L. pneumophila* are not restricted to the T4SS. It is posited that bacterial effectors have been acquired by both HGT and convergent evolution [207]. We favour the hypothesis that eukaryotic-like T2SS effectors were acquired via HGT, as HGT is detectable at the primary sequence level, whereas convergent evolution is more commonly detected at the gross structural level [203]. Given that only a few annotated genomes of protozoa exist yet amoebae are probably major contributors to HGT, our understanding of the origins of these eukaryotic-like *Legionella* proteins is only just beginning. Furthermore, we suspect that the numbers of eukaryotic/protozoan-like T2SS substrates will rise substantially as more amoebal genomes are sequenced.

### Novel effectors

Interestingly, 27 of T2SS effectors encoded by *L. pneumophila* do not share significant structural or sequence similarity to any known enzyme(s). Seven of these novel effectors (i.e. NttA, NttB, NttC, NttD, NttE, NttF and NttG) have been validated as T2S substrates, i.e. they are present in wild-type supernatants but not *lsp* mutant supernatants (Table 1). The other 20 (i.e. Lpg0042, Lpg0165, Lpg0198, Lpg0301, Lpg0374, Lpg798, Lpg0957, Lpg1030, Lpg1233, LvrE, Lpg1318, Lpg1431, Lpg1645, Lpg1647, WipC, Lpg2220, Lpg2246, Lpg2275, Lpg2320 and Lpg2443) have been detected in wild-type strain Philadelphia-1 supernatants but have not yet been examined for their lack of secretion by the corresponding *lsp* mutant (Table S2). In many cases, members of this class of T2SS substrates share, to varying degrees, sequence similarity to hypothetical proteins in other bacteria. For example, NttD, possessing the conserved domain of unknown function (DUF) 4785, has homologues that are found primarily in phylogenetically related *G* *amma* *proteobacteria*. On the other hand, NttB has putative homologues only among aquatic *Piscirickettsia* species and *Silvanigrella aquatica*. Recent structural and biochemical analysis revealed that NttB is a C1 family peptidase that diverged from common papain‐like cysteine proteases and forms a distinct phylogenetic lineage from eukaryotic cathepsins [152]. NttF has only a single homologue found in the genome of *Piscirickettsia litoralis*. Finally, NttG has only a single homologue, and that related protein is encoded by aquatic *Francisella halioticida*, a member of a genus that, like *Legionella*, is pathogenic for both amoebae and humans [208, 209]. Structural analysis suggests that NttG is a VirK-like protein, yet its activity and role of infection remain undefined [154]. Arguably most interestingly, some members of this general class of T2S substrates do not have any putative homologues (E-value <1×10^−10^) outside of the genus *Legionella*, further suggesting that many of the T2SS-dependent proteins may be highly specialized for the intra-amoebal lifestyle of *Legionella* species [25, 113]. These include the documented T2SS substrates NttA, NttC and NttE (Table 1) as well as the putative substrates Lpg0042, Lpg0374, Lpg0798, Lpg1233 and Lpg2443 (Table S2). Importantly, both of the effectors in this category that have been assessed, using mutant analysis, for their role in intracellular infection were found to be required for optimal growth within amoebae. Whereas NttA is necessary for infection of *A. castellanii* and *W. magna*, NttC is required for infection of *V. vermiformis* and *W. magna* [25, 113]. Given the novelty of NttA and NttC, it is difficult predict how these T2S substrates promote intracellular infection; however, further phenotypic analysis of the *nttA* and *nttC* mutants as well as biochemical and structural analysis of the NttA and NttC proteins may represent fruitful lines of inquiry. Although not peculiar to the genus *Legionella* because of related hypothetical proteins occurring mainly in *G* *amma* *proteobacteria*, the novel effector NttD is also required for optimal infection of *A. castellanii* [76]. The structure of NttD has been recently obtained; but, unfortunately, this information has not yet provided a strong clue as to the activity of NttD [76]. Given that three of four novel effectors examined (i.e. NttA, NttC and NttD; but not NttB) promote infection of at least one amoebal host, it is likely that the emergence of novel T2SS substrates plays a significant role in both the ecology and the pathogenesis of *L. pneumophila*.

### Transcriptional analysis and regulation of T2SS effectors

Recently, qRT-PCR analysis was used to assess the relative expression of 19 of the 25 known effector genes during multiple stages of *L. pneumophila* growth in bacteriological media as well as during intracellular replication in three amoebae and human macrophages [76]. The T2SS substrate genes showed a range of expression patterns as opposed to displaying similar responses to the various growth environments; for example, eight of the 19 genes were up-regulated upon intracellular infection, and eight others were down-regulated [76] (Table S3). Together, these data imply that the amounts of proteins that are elaborated by the T2SS are dictated, at least primarily, at the level of the individual effector-gene or of subsets of effector-gene transcription as opposed to being controlled at the level of T2SS apparatus gene transcription or by a single global regulator that acts upon the many effector genes [76]. In further support of this conclusion, earlier studies had found that *celA, chiA, legP*, *map* and *nttA* are modulated by the regulators PmrA and PmrB [210], whereas *lipA* and *lipB* are influenced by LetA and RpoS [129], and *gamA* is affected by CsrA [211]. The CpxRA two‐component system, which controls expression of the Dot/Icm system and effectors, was also shown to positively regulate expression of 13/25 T2SS effectors [212], including at least six factors (LapA, NttA, NttD, PlaC, ProA and SrnA) that promote intracellular replication in protozoa (Table 1). A comprehensive summary of the various regulatory aspects of the T2SS effector genes is presented in Table S3.

Although proteomics and ensuing mutant analysis has been the principal means by which T2SS-dependent proteins that promote infection have been identified, transcriptional profiling has recently been shown to be a valid alternative. Indeed, the importance of LapA and PlaC for infection of *A. castellanii* was revealed through a novel combination of transcriptional and mutational analyses [76]; that is, (i) the two genes were first found to be among the most up-regulated effector genes during wild-type infection of the amoebae, (ii) transcript profiling of a *lapA* mutant then showed even higher levels of *plaC* mRNA, and conversely a *plaC* mutant exhibited elevated levels of *lapA* transcription, and (iii) a newly made, double mutant lacking both *lapA* and *plaC* exhibited a loss of infectivity, uncovering redundant yet important roles for LapA and PlaC in nutrient acquisition and intracellular bacterial growth.

## The T2SS and its effectors belong to the core genome of *L. pneumophila*

Although the vast majority of studies on the T2SS have utilized serogroup (SG)−1 strain 130b and to a lesser extent SG-1 strains Philadelphila-1 and Corby, we and others previously reported the presence of T2SS apparatus proteins encoded in a variety of clinical and environmental *L. pneumophila* isolates [42, 76, 213–215]. Extending this analysis to all of the 90 annotated *L. pneumophila* complete genome assemblies currently in the NCBI Reference Sequence (RefSeq) Database [216], encompassing eight of the 15 SGs, we found that all of the T2SS apparatus genes are intact in all of the strains, except for frame-shift mutations in *lspK* in SG1 strain Flint 2 (D-7477), *lspL* in SG1 strain FFI103 and *pilD* in SG1 strain L10/23. The minimum amino-acid identity of the Lsp homologues compared to the *L. pneumophila* 130b Lsp proteins was 93.5 % for LspC, 96.5 % for LspD, 97.0 % for LspE, 96.5 % for LspF, 98.6 % for LspG, 90.2 % for LspH, 92.8 % for LspI, 93.7 % for LspJ, 91.9 % for LspK, 88.7 % for LspL, 94.2 % for LspM and 89.2 % for PilD/LspO, in agreement with our previous findings from analysing a panel of 17 strains [76]. Some apparatus proteins such as LspJ may undergo diversifying selection within *L. pneumophila* [217], which may help to explain the varying degrees of secreted activity of environmental isolates despite encoding an intact T2SS [218]. Turning attention to the prevalence of the secreted substrates, it is clear that the genes for all 25 confirmed T2SS effectors (Table 1) are present and intact within the 90 annotated *L. pneumophila* genomes, with the sole exception being a frame-shift mutation in *gamA* in SG1 strain Albuquerque 1 (D-7474). Overall, these findings suggest that the T2SS along with many effectors belongs to the core genome of *L. pneumophila*. While the T4SS also belongs to the core genome of *L. pneumophila*, it has been reported that up to 30 % of T4S effectors belong to the accessory genome and undergo increased rates of pseudogenization [89].

## Conservation of the T2SS apparatus genes within *Legionella*

### Description of T2SS genes in other *Legionella* species

**Fig. 5.**
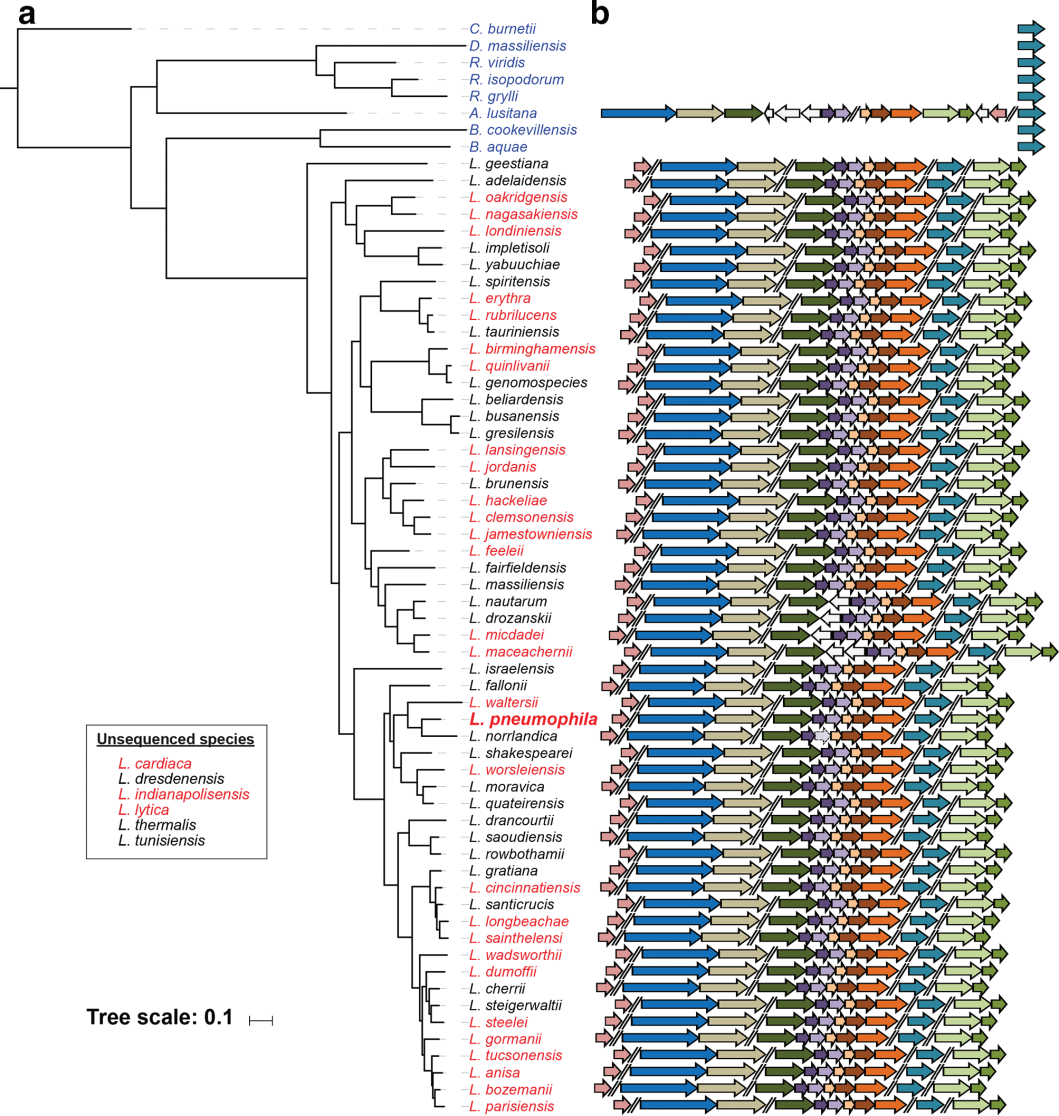
Phylogenetic analysis and distribution of T2SS genes in *Legionellales*. (a) A list of all currently named *Legionella* species, their phylogenetic relationships based upon data from whole-genome sequencing, and their association with human disease. A maximum-likelihood phylogenetic tree was constructed in RaxML (LG+Γ+F model) [111] from the concatenated amino acid sequences derived from 78 near-universal single-copy genes [228]. Support values >50 (from 100 bootstrap replicates) are given at the corresponding nodes. Bar, 0.1 amino acid substitutions per site. *Legionella* species coloured in red have been associated with human disease, and those in black have not (yet) been linked to disease. Appearing at the top of the list are non-*Legionella* species (blue) that belong to other genera within the *Legionellales*. (b) A depiction of the distribution of the 12 core *lsp* T2SS genes (represented by coloured arrows as in Fig. 1) throughout the order *Legionellales*. Distinct genetic loci are separated by double slashes. White arrows indicate genes unrelated to the Lsp T2SS. Arrows filled with hatch marks indicate pseudogenes. Gene arrows are drawn to scale.

As mentioned in the introductory section, 63 species of *Legionella* have thus far been characterized, with 32 of them already being linked to human disease (Fig. 5a). Moreover, whenever examined, the non-*pneumophila* species have also proven to be intracellular parasites of amoebae [11, 219–224]. Nonetheless, our understanding of these *Legionella* species, including *L. longbeachae*, which is the most prevalent disease-causing species in Australia, has lagged very far behind that of *L. pneumophila* [14, 20, 224, 225]. The presence of T2SS genes in non-*L. pneumophila* species of *Legionella* was first detected in *L. cherrii*, *L. feeleii*, *L. gormanii*, *L. longbeachae*, *L. micdadei*, *L. parisiensis* and *L. spiritensis* by Southern blot analysis [42], prior to the sequencing of any of the non-*pneumophila* species [224, 226]. With the elucidation of many *Legionella* species genomes, we previously confirmed the presence of the T2SS apparatus genes among all 41 *Legionella* species examined [76]. Extending this analysis to include all 57 of the currently sequenced *Legionella* genomes, coding sequences corresponding to all core T2SS genes are present across the genus (Fig. 5b). While all *lsp* genes were detected in all of the 57 species analysed, there were two notable differences in the gene arrangements. First, there were intergenic insertions between *lspF* and *lspG* in the *lspFGHIJK* locus in *L. drozanskii*, *L. maceachernii*, *L. micdadei* and *L. nautarum* (Fig. 5b). All species in this clade had inserted a gene that encodes TesA, a signal sequence-containing, multi-functional periplasmic protein with thioesterase 1/protease 1/lysophospholipase L1 activity [227]. Since *L. drozanskii*, *L. maceachernii*, *L. micdadei* and *L. nautarum* are monophyletic (Fig. 5a), the insertion event probably occurred once and has persisted since. Based on blastp analysis of TesA, the source of *tesA* was likely aquatic bacteria including *Polynucleobacter* or *Vibrio* spp., and no TesA homolog was found to occur within the *L. pneumophila* genome. *L. maceachernii* also had a gene encoding a hypothetical protein inserted between *lspF* and *tesA* (Fig. 5b). This putative protein lacks a secretion signal and is found strictly within *L. maceachernii* based on the absence of any homologues in the blast protein database. As *lspG* transcription is not linked to *lspF* (Fig. 1b), insertion of genes between *lspF* and *lspG* is unlikely to impact transcription from the pseudopilin gene operon (*lspGHIJK*). The second notable difference regarding the *lsp* genes among the *Legionella* genus is the apparent pseudogenization of the *lspH* gene of *L. norrlandica* resulting in a truncated LspH protein. Although most closely related to *L. pneumophila* (Fig. 5a), *L. norrlandica* is avirulent in a protozoan infection model and is unable to establish large replication vacuoles [11]. Inactivation of the *lspGHIJK* pseudopilin gene cluster in *L. pneumophila* results in loss of T2S activities in culture supernatants and inability to grow within protozoa [39]. Thus, we hypothesize that the attenuation of *L. norrlandica*, which incidentally possesses an intact T4SS [11], is attributable to loss of the T2SS via the *lspH* mutation.

### Conservation and context of T2SS genes among *Legionella* species

The degree of conservation of Lsp protein sequences is more variable among the *Legionella* species than it is amongst *L. pneumophila* strains. The major pseudopilin LspG displays the highest degree of conservation, with a minimum amino-acid identity of 78.8 % among species relative to LspG in *L. pneumophila*. The minimum amino-acid identity for other Lsp proteins is 77.3 % for LspE, 70.6 % for LspF, 63.1 % for LspD, 54.4 % for LspI, 51.5 % for LspJ, 48.2 % for PilD/LspO, 41.8 % for LspH, 40.3 % for LspC, 40.0 % for LspK, 37.8 % for LspM and 34.6 % for LspL. Despite the higher divergence among sequences, only LspD has reportedly undergone diversifying selection within certain clades of the *Legionella* evolutionary tree [217], which may play a role in diversification of T2SS function, and consequently defining the ecological niche of *Legionella* species.

**Fig. 6.**
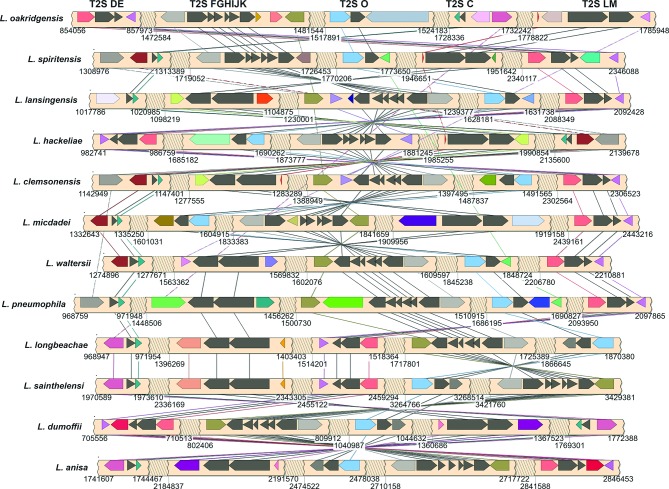
Chromosomal organization of the T2SS genes in different *Legionella* species. The genetic context of the five *lsp* gene clusters within 12 fully sequenced *Legionella* species was determined using SimpleSynteny [230]. Dark grey arrows depict *lsp* genes. Other coloured arrows represent genes flanking the *lsp* gene clusters. Orthologous genes are joined by vertical lines. The genomic coordinates are given beneath each genome segment.

The chromosomal organization of *lsp* gene clusters varies across the genus. Currently, at least 12 different arrangements of the *lsp* gene clusters are evident (Fig. 6). Each arrangement is most similar among phylogenetically close species, such as *L. waltersii* and *L. pneumophila*. Although the five *lsp* gene clusters are found intact across all *Legionella* species, the genetic context of each cluster varies. In some cases, *lsp* genes are flanked by conserved orthologous genes; for example, T2S O (*pilD*) was always found as the last gene in the *pilBCD* operon, and T2S C (*lspC*) was always immediately upstream of a Sel1 repeat-containing protein. In other cases, the flanking genes were highly variable. The genes flanking the *lspDE* cluster were notable for being highly diverse among the analysed *Legionella* species. In *L. spiritensis*, *L. hackeliae* and *L. clemsonensis*, there was a methionine tRNA immediately upstream of T2S D. In *L. oakridgensis*, an ISL3 family insertion sequence has transposed between the tRNA and T2S D. It is well established that tRNAs serve as integration sites in various prokaryotes [229]. In *Legionella* species, the type IVA secretion system is encoded on a plasmid-like element that integrates at the 3' end of various tRNAs in both *L. pneumophila* and *L. longbeachae* [226]. Thus, the close proximity of the *lspDE* gene cluster to a tRNA gene may explain the high diversity of flanking genes observed. Given the various arrangements of the five *lsp* gene clusters on each chromosome, it is clear that extensive chromosomal rearrangements have occurred throughout the evolution of *Legionella* species, as has been previously described [217, 224].

## Distribution of T2SS substrates across the genus *Legionella*

### General patterns and distributions for specific substrates

**Fig. 7.**
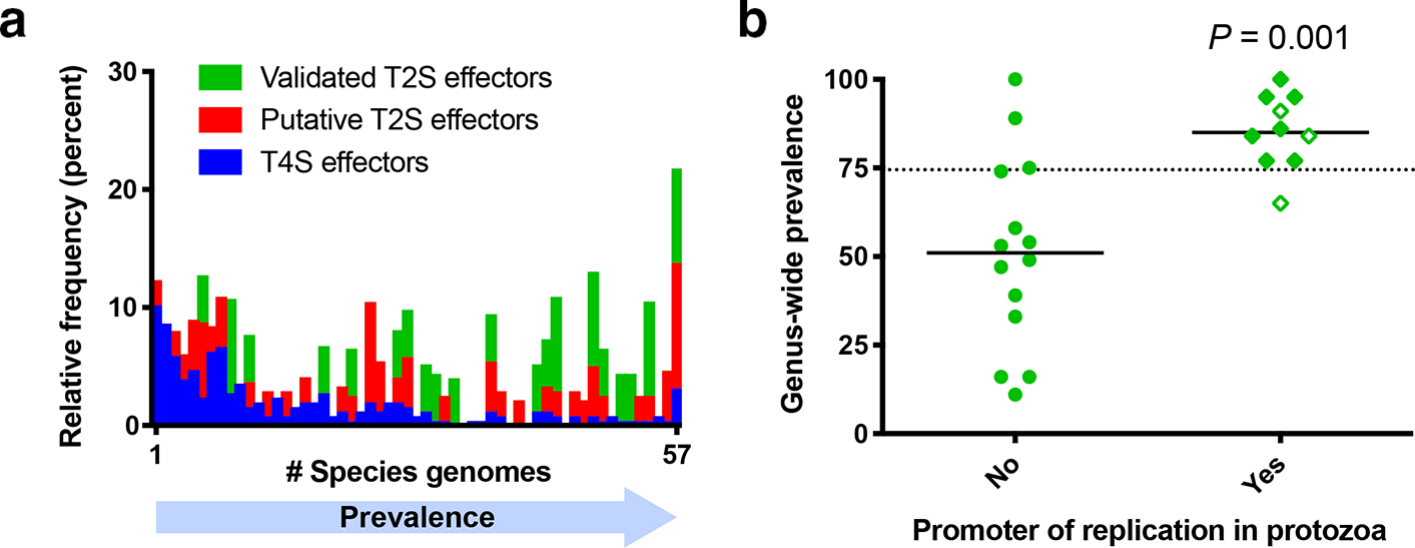
The genus-wide prevalence of *L. pneumophila* effectors. (a) The distribution of documented Lsp T2SS effectors (*N*=25), putative T2SS effectors (*N*=47), and a subset of documented Dot/Icm T4SS effectors (*N*=255) among the 57 analysed *Legionella* species was determined. The relative frequency of effector groups (*y*-axis) was determined by the number of species genomes in which individual effectors were present (*x*-axis), with *L. pneumophila*-specific effectors at the far left (i.e. *x*=1) and core effectors at the far right (i.e. *x*=57). (b) The role of validated T2SS effectors in protozoan infection versus the genus-wide prevalence was determined for *N*=24 experimentally characterized T2SS effectors based on mutant analysis in a protozoan infection model. Open symbols represent those effectors for which genetic complementation has not yet been achieved. A Student’s *t*-test was performed between the two sample distributions. The dashed line represents the median genus-wide prevalence for the analysed effectors.

The 25 T2SS substrates that have been confirmed for *L. pneumophila* strain 130b exhibit a range of distributions across the genus *Legionella* (Table 1), reinforcing an earlier conclusion that was based on the prevalence of LapA, NttD, PlaC and ProA within *Legionella* [75]. Extending this analysis to include both the 25 validated and the 47 putative T2SS substrates, it appears that the vast majority of T2SS effectors associated with *L. pneumophila* are found in 32 or more of the 57 *Legionella* species analysed (Fig. 7a). This stands in marked contrast to the situation for the Dot/Icm T4SS, where the majority of a subset of T4SS effectors analysed (*N*=255) are found in only nine or fewer of the *Legionella* species (Fig. 7a) [196, 229]. Moreover, seven out of the 72 T2SS (documented+putative) effectors (9.7 %) are conserved in all 57 species and thereby represent ‘core’ effectors. Once again, this level of conservation is rather different from the T4SS where there are only eight core effectors out of 255 T4SS effectors examined (3.1 %) [196, 229]. There appears to be only one *L. pneumophila*-specific T2SS effector, namely the putative effector Lpg0165 (Table S2). This presents yet another distinction from the Dot/Icm system, where at least 20 T4SS effectors are *L. pneumophila*-specific [229]. In summary, a majority of the T2SS effectors appear to be shared by a large subset of *Legionella* species, hinting at a critical role for them in the ecology of *Legionella* owing to their long evolutionary history across the genus.

**Fig. 8.**
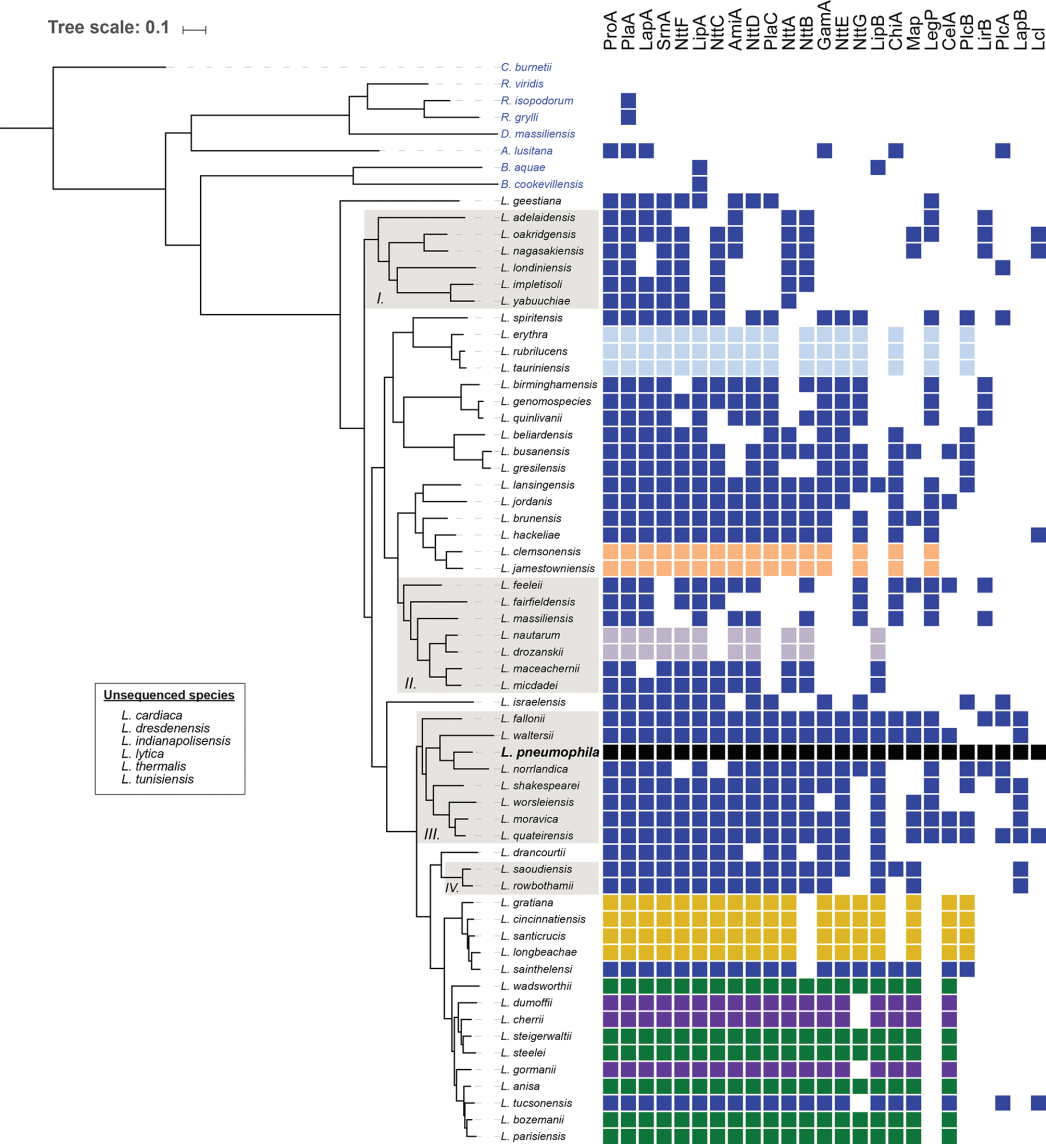
Distribution of T2SS substrates across the genus *Legionella* and beyond. The presence/absence of the 25 Lsp T2SS substrates in all sequenced members of the order *Legionellales* was determined using blastp as previously described [76]. Black cells indicate the presence of all 25 substrates in the *L. pneumophila* genome. Rows of the same colour (with the exception of dark blue) indicate effector repertoires shared by more than one *Legionella* species. Selected clades undergoing effector gain/loss are highlighted in grey and numbered. Bar, 0.1 amino acid substitutions per site.

Turning attention specifically to the distribution of the 25 known T2SS effectors (Table 1), the metalloprotease ProA and the phospholipase A/lysophospholipase A PlaA are notable for being found in all 57 species analysed, and thus constitute the first examples of ‘core’ effectors of the *Legionella* T2SS (Fig. 8). While only ProA and PlaA are found within all genomes, the acylglycerol lipase LipA, aminopeptidase LapA, novel effector NttF and ribonuclease SrnA were found in 89–95 % of the *Legionella* species (Fig. 8). Interestingly, LipA and ProA are found immediately adjacent to one another within the *L. pneumophila* chromosome (Fig. 3), and the rate of co-occurrence and synteny in the genome was 89 % among the 57 *Legionella* species analysed. Since effector genes encoded within close proximity in the *L. pneumophila* genome may coordinate their functions or regulate one another [231, 232], ProA and LipA might function in a coordinated fashion. Six other effectors, i.e. phospholipase A PlaC, putative amidase AmiA, and the novel effectors NttA, NttB, NttC and NttD, were found in 75–86 % of species (Fig. 8). Of the 12 effectors with >75 % conservation, seven (i.e. LapA, NttA, NttC, NttD, PlaC, ProA and SrnA) clearly promote infection of at least one protozoan host [25, 76, 113, 158]. Additionally, preliminary studies suggest that AmiA and novel effector NttF may also promote infection of protozoa [140, 153], potentially bringing the total to nine out of 12. Although *plaA* mutants have thus far not been shown to be impaired in infection, PlaA appears to influence the integrity of the LCV membrane, with a mutant lacking the T4SS effector SdhA producing a highly unstable LCV, and this loss of LCV membrane integrity was reversed upon subsequent mutation of PlaA [156]. Thus, the vast majority of the known T2SS effectors that are highly prevalent within the genus are implicated in *L. pneumophila* infection of amoebae. In contrast, nine T2SS effectors (i.e. CelA, ChiA, GamA, LegP, LipB, Map, NttE, NttG, PlcB) have a prevalence of 33–74 % within the *Legionella* species (Fig. 8), and none of them are yet clearly implicated in infection (Table 1). Finally, four effectors, i.e. LapB, Lcl, LirB and PlcA, were found in fewer than 20 % of *Legionella* species (Fig. 8). LapB and PlcA are dispensable for infection of protozoa [25, 143], as noted above, whereas Lcl promotes attachment and invasion in the protozoa *V. vermiformis* and *A. castellanii* but not intracellular growth per se [116]. Overall, this analysis indicates that the T2SS effectors known to be involved in intracellular infection of at least one eukaryotic host are significantly more prevalent throughout *Legionella* as compared to those effectors that are not required for intracellular infection of natural host cells (Fig. 7b). Thus, we hypothesize that T2SS effectors that are more prevalent within the genus *Legionella* are under stronger selective pressure due to their role in infection of the natural host.

### Groupings amongst the *Legionella* species based on their T2SS substrates

Although most of the documented T2SS effectors are widely distributed across the genus *Legionella*, no other *Legionella* species analysed possessed the same effector repertoire that was found in *L. pneumophila* (Fig. 8). *L. quateirensis*, *L. fallonii* and *L. waltersii* were most similar to *L. pneumophila* in this regard with each having 21 or 22 of the effectors (Fig. 8). *L. longbeachae*, the second most common cause of Legionnaires’ disease, has 18 of the effectors (Fig. 8). *L. geestiana*, *L. fairfieldensis*, *L. londiniensis*, *L. adelaidensis*, *L. impletisoli* and *L. yabuuchiae*, the species most distantly related to *L. pneumophila*, possessed the lowest number of shared effectors at 7–10 (Fig. 8). Some of the species examined had shared subsets of the T2SS substrates, and there appeared to be six distinct groupings of species based upon these shared effectors. The first group included *L. wadsworthii*, *L. steigerwaltii*, *L. anisa*, *L. bozemanii*, *L. parisiensis* and *L. steelei*, which lack LapB, Lcl, LegP, LirB, PlcA and PlcB (green cells in Fig. 8). While this effector repertoire is found among phylogenetically related *Legionella* species, other effector repertoires are interspersed within this clade. Thus, perhaps the repertoire shapes the environmental niche, and is not simply defined by the evolution of the genus. The second group included *L. cherrii*, *L. dumoffii* and *L. gormanii*, which lack LapB, Lcl, LegP, LirB, Lpw18641, PlcA and PlcB (purple cells in Fig. 8). Like the first repertoire, *L. cherrii* and *L. dumoffii* are paraphyletic, while *L. gormanii* belongs to a different phyletic group; thus, these species may also occupy another environmental niche. The third group included *L. gratiana*, *L. cincinnatiensis*, *L. santicrucis* and *L. longbeachae*, which lack ChiA, LapB, Lcl, LegP, LirB, NttB and PlcA (gold cells in Fig. 8). All four of these species are paraphyletic, and thus this repertoire probably arose from divergent evolution (i.e. gene gain and loss) within a single *Legionella* clade. The fourth group was the monophyletic group of *L. rubrilucens*, *L. erythra* and *L. tauriniensis*, which lack NttA, LipB, Map, CelA, PlcA, LapB, LirB and Lcl (light blue cells in Fig. 8). The fifth was the monophyletic group of *L. jamestowniensis* and *L. clemsonensis*, which lacked Lpw_02811 (Lpg0189), LipB, Map, CelA, PlcA, PlcB, LapB, LirB and Lcl (beige cells in Fig. 8). The final group was the monophyletic group of *L. nautarum* and *L. drozanskii*, which lacked NttC, PlaC, GamA, NttE, NttG, ChiA, Map, LegP, CelA, PlcA, PlcB, LirB, LapB and Lcl (grey cells in Fig. 8). All other *Legionella* species possess ‘unique’ repertoires, which, overall, range in size from 25 in *L. pneumophila* (black cells) to seven in *L. yabuuchiae*. Overall, this comparison of the T2SS effector repertoires across the 57 examined *Legionella* species suggests that phylogenetically more closely related species share similar sets of effectors. However, this *L. pneumophila*-centric view of T2SS effectors may underestimate the true number and distributions of *Legionella* T2SS effectors, as there are T2SS-compatible extracellular activities that are not dependent on the 25 known *L. pneumophila* effectors, as noted above. Furthermore, it is entirely possible that there are T2SS effectors expressed by non-*pneumophila* species that are absent from *L. pneumophila*. In this vein, it is worth noting that there are >600 orthologous T4SS effectors across the genus *Legionella*, approximately half of which are absent from *L. pneumophila* [228].

At least 15 examined species of *Legionella* beyond *L. pneumophila* are known to possess secreted activities compatible with T2S. Given that homologues of ProA are found among all sequenced *Legionella* genomes, it is not surprising that *L. anisa*, *L. cincinnatiensis*, *L. dumoffii*, *L. erythra*, *L. feeleii*, *L. gormanii*, *L. jordanis*, *L. longbeachae*, *L. moravica*, *L. parisiensis, L. steigerwaltii* and *L. wadsworthii* all secrete protease activity [41, 233, 234]. In a similar way, it is logical that a phospholipase A activity, robably due to PlaA, has been detected in supernatants from *L. anisa*, *L. dumoffii*, *L. gormanii*, *L. jordanis*, *L. longbeachae*, *L. oakridgensis*, *L. parisiensis* and *L. steigerwaltii* [233, 235]. Moreover, *L. dumoffii*, *L. gormanii* and *L. steigerwaltii* supernatants are positive for acid phosphatase activity, compatible with their genomes’ encoding homologues of Map (Fig. 8) [233]. *L. longbeachae*, *L. bozemanii*, *L. dumoffii*, *L. gormanii*, *L. jordanis* and *L. micdadei* all possess secreted phospholipase C activity [236]. However, only *L. longbeachae* encodes a homologue of either PlcA or PlcB, and although *L. erythra* secretes an endoglucanase activity [141], it lacks a homologue of CelA (Fig. 8). These latter observations lend support to the view that there are additional T2SS effectors within the genus *Legionella* that are not present within *L. pneumophila*.

### Examples of how T2SS substrates are gained and lost within the genus

Based on the distribution patterns summarized above, a recent study proposed possible scenarios by which select T2SS substrates were gained and lost within the genus *Legionella* [76]. The first substrate, PlaC, appears to be an ‘ancestral’ T2SS effector that has undergone two loss events over time, once in clade ‘I’ and once in clade ‘II’ (Fig. 8, grey shaded regions). The NttD substrate is another ancestral T2SS effector, but one that seems to have undergone three loss events: once in clade I (Fig. 8, grey shaded region), once in *L. fairfieldensis* and once in *L. drozanskii*. LapA appears to be a second ancestral T2SS effector that has undergone three loss events: once in *L. nagasakiensis*, once in *L. londiniensis* and once in *L. maceachernii*. On the other hand, LapB probably arose from a recent gene duplication of LapA, having occurred twice: once in clade ‘III’ (and subsequently lost in *L. norrlandica*) and once in clade ‘IV’ (Fig. 8, grey shaded regions). This second gene copy probably underwent positive selection and emerged with a new substrate specificity that is non-redundant with the closely related LapA [76, 143].

## On the origins of the *Legionella* Lsp T2SS

**Fig. 9.**
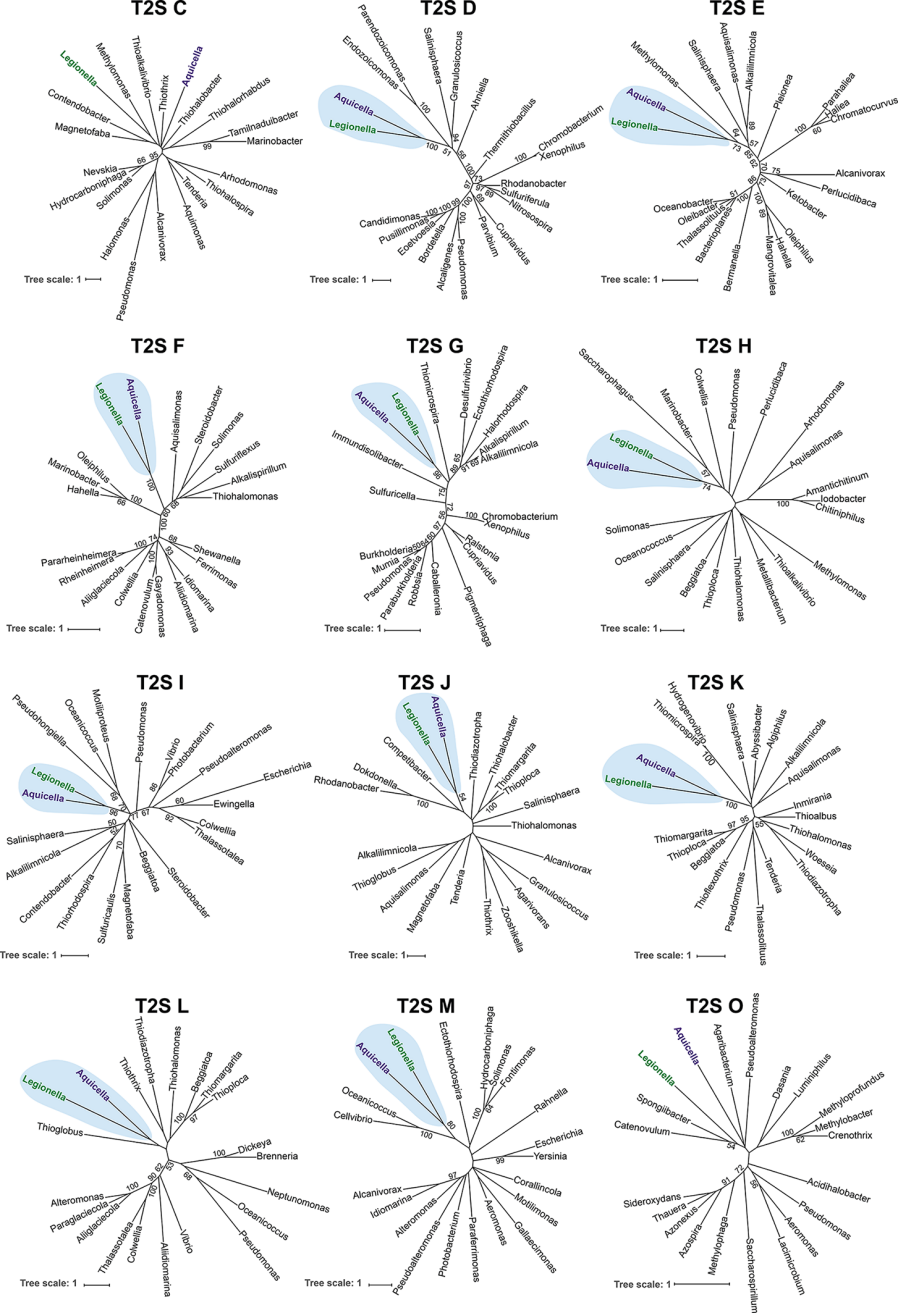
Phylogenetic analysis of Lsp T2SS proteins of *Legionella*. Homologes of *Legionella* T2SS apparatus proteins were identified by blastp using a minimum query coverage of 60 % for T2S DEFGHIJKLMO or 30 % for T2S C, and amino acid identity and E-value cutoffs of 30 and 1×10^−10^, respectively, for T2S DEFGIO, and amino acid identity and E-value cutoffs of 20 and 1×10^−5^, respectively, for T2S CHJKLM. Maximum-likelihood trees were generated from full-length amino acid alignments of all 12 individual T2SS apparatus proteins (i.e. T2S CDEFGHIJKLMO) and the most closely related homologues (as determined by blastp) encompassing 20 unique genera using RaxML (100 bootstrap replicates, GTR + Γ model) [111]. Bootstrap support values >50 are presented at the respective nodes. Bar, 0.1 amino acid substitutions per site. Labels representing *Legionella* Lsp proteins are in bold green, and labels representing *Aquicella* proteins are in bold purple. Monophyletic clades containing only *Legionella* and *Aquicella* T2SS orthologues are shaded in blue.

*Legionella* species (the sole members of the family *Legionellaceae*) are most closely related to members of the family *Coxiellaceae* which contains *Coxiella*, *Rickettsiella*, *Aquicella, Berkiella, Occultobacter* and *Nucleophilum* among others*,* which together with *Legionellaceae* make up the order *Legionellales* [3]. It was previously reported that *Coxiella burnettii*, although possessing a T4SS, lacks a core set of T2SS genes [50, 237]. Therefore, it had remained unclear when the *Legionella* T2SS emerged within the *Legionellales*, if at all within the closely related *Coxiellaceae*. When the additionally available *Coxiellaceae* genomes encompassing three species of *Rickettsiella* [RefSeq assembly accessions GCF_001881485.1 (*Rickettsiella grylli*), GCF_000168295.1 (*R. grylli*), GCF_003966755.1 (*Rickettsiella viridis*) and GCF_001881495.1 (*Rickettsiella isopodorum*)], one species of *Diplorickettsia* (RefSeq assembly accession GCF_000257395.1 (*Diplorickettsia massiliensis*)], and two of *Berkiella* [RefSeq assembly accessions GCF_001431295.1 (*Berkiella aquae*) and GCF_001431315.1 (*Berkiella cookevillensis*)] were examined, there was also no evidence of T2SS apparatus genes, other than *pilD*/*lspO*, which was linked, as is often the case, to other T4P genes. However, further analysis revealed a complete set of T2SS components within the genome of the very recently sequenced *A. lusitana* (RefSeq assembly accession GCF_003350455.1), an aquatic bacterium within the *Coxiellaceae* [196]. Moreover, five of 12 core Lsp proteins from *L. pneumophila* (i.e. LspD, LspF, LspI, LspK and LspL) shared their highest amino-acid identity with the orthologous T2SS components of *A. lusitana*, with identity ranging from 24 % for LspL to 49 % for LspF (Fig. 9). All of the other seven Lsp proteins also showed strong sequence relatedness to their *Aquicella* counterparts, although their closest homologues existed in various other types of *G* *amma* *proteobacteria*. Phylogenetic analysis provided further evidence that the majority of Lsp proteins (10 of 12) are most closely related to proteins in *A. lusitana*. (Fig. 9). In the case of LspC, due to the high sequence divergence among related T2S C proteins, the evolutionary history could not be reliably inferred, with very few branches containing bootstrap values >50. In the case of PilD, the branch was adjacent to that of *A. lusitana* T2S O; however, it was not monophyletic but intermediate between a clade containing *Aquicella* and a clade containing *Spongiibacter*, which has also been isolated from protozoa [238]. Given the dual role of T2S O in both protein secretion and T4P biogenesis, and that T4P are present in all members of the order *Legionellales*, the evolutionary trajectory of T2S O is not as clear. Therefore, it appears that the T2SS apparatus of *Legionella* is closer to that of *Aquicella* than to any other bacterial genus. Whereas the environmental reservoirs of the obligate intracellular bacteria belonging to *Coxiella* and *Rickettsiella*/*Diplorickettsia* are thought to be primarily mammals and arthropods, respectively [239–241], *Aquicella* like *Legionella* can be routinely cultured in the laboratory and replicates intracellularly within aquatic protozoa [196, 242]. Interestingly, *Berkiella* species are the closest relative to *Legionella* yet are obligate intracellular parasites of amoebae and replicate inside the host cell nucleus [243]. Thus, we posit two scenarios for the emergence of the *Legionella* Lsp T2SS: the Lsp-like T2SS apparatus emerged within the *Legionellales* in a common ancestor shared between *Rickettsiella–Diplorickettsia–Aquicella–Berkiella–Legionella*, and was lost twice, once in the *Rickettsiella–Diplorickettsia* clade and once in the *Berkiella* clade; alternatively, the Lsp-like T2SS apparatus emerged within *A. lusitana*, and was subsequently acquired in a *Legionella* progenitor via HGT within protozoa. While it is unknown whether the T2SS contributes to the ability of *Aquicella* to replicate within the protozoa (in the cytosol, or at minimum not intranuclear) and outside of the host, we posit that this is the case based upon the compelling importance that T2SS has in the intracellular parasitism and extracellular survival and persistence of *L. pneumophila*. Intriguingly, the *A. lusitana* genome possesses homologues of six of the 25 known *L. pneumophila* T2SS effectors, including ProA (E=1×10^−143^), PlaA (E=2.16×10^−45^), GamA (E=5.3×10^−65^), LapA (E=1.0×10^−113^), PlcA (E=3.20×10^−136^) and ChiA (E=1.12x10^−150^), a pattern that is not too dissimilar from that of *L. londiniensis* and *L. adelaidensis*, both of which have nine out of the 25 (Fig. 8). The fact that at least ProA, PlaA and LapA have a role in intracellular infection by *L. pneumophila* also further implies an importance for T2SS in the intracellular parasitism and ecology of *Aquicella*. Interestingly, five of the *L. pneumophila* effectors had their closest homologue occurring in *A. lusitana*, more than for any other genus (Table 1). In contrast, current blastp analysis found no orthologues to any of the known T2SS effectors of *Legionella* in *C. burnetii*, *D. massiliensis* and *R. viridis*, and only a couple in species of other *Rickettsiella* or *Berkiella* genomes (Fig. 8). Given the complete lack of T2SS genes in *C. burnetii*, we hypothesize that the T2SS emerged after the divergence of *Coxiella* species from other *Coxiellaceae* (and *Legionellaceae*) members. In summary, based upon the latest updates in the genome database, we suggest that the T2SS of *L. pneumophila* originated from within the order *Legionellales* and that many of the effectors may have also arisen within that progenitor.

## Concluding thoughts

That the *L. pneumophila* T2SS, with its 25 validated substrates (Table 1), has a major role in the ecology and pathogenesis of the Legionnaires’ disease agent is now well known, as summarized in the initial sections of this review. Looking to the future, it will be instructive to confirm whether the many putative substrates of the *L. pneumophila* T2SS (Table S2) are *bona fide* substrates. Such a finding would clearly document that the output of a T2SS can be quite large and varied, perhaps encompassing a wealth of novel enzymes. In the meantime, it will be important to more precisely define the enzymatic activities and molecular modes of action of the known T2SS substrates, particularly those that are required for the ability of *L. pneumophila* to infect host cells, evade immune defences or mediate damage to tissue. Happily, substrates of the *Legionella* T2SS have recently garnered the attention of structural biologists leading to the reporting of nine crystal structures (Tables 1 and S2). Further expansion in this dataset will probably enhance both our understanding of substrate activities and the mechanism of the secretion process itself, including elucidating how the 3-D structures of the substrates are recognized by the secretion apparatus. Another fruitful area for future investigation is delving more deeply into the regulatory networks that control the expression of the T2SS apparatus and/or its many different effectors. The available data (Table S3) already indicate that the regulation of T2SS function is complex and multifactorial; nonetheless, deciphering how the activity of the T2SS is coordinated with that of the Dot/Icm T4SS and other systems is critical for understanding the overall virulence strategy of the *Legionella* pathogen.

The rapidly expanding number of genome sequences available for the genus *Legionella* and beyond has greatly facilitated our understanding of the distribution and diverse origins of the T2SS and its arsenal of effectors, as presented in the latter part of this review. It is now clear that the genes encoding the T2SS apparatus are absolutely conserved across the genus *Legionella*, which includes 62 species and more than 30 pathogens in addition to *L. pneumophila*. Moreover, the vast majority of the T2SS effectors associated with *L. pneumophila* are shared by a large number of other *Legionella* species, signalling at a key role for them in the ecology of *Legionella* as a whole. However, no other species has the same effector repertoire as does *L. pneumophila*, with, as a general rule, phylogenetically more closely related *Legionella* species sharing similar sets of effectors. Interestingly, the T2SS effectors that are involved in intracellular infection of a eukaryotic host(s) are significantly more prevalent throughout *Legionella*, indicating that they are under stronger selective pressure.

**Fig. 10.**
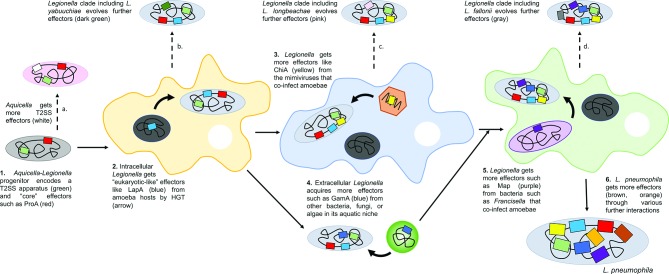
Model for evolution of T2SS and its effectors within the genus *Legionella*. The acquisition of T2SS effectors over (evolutionary) time, with the last common ancestor among *Legionella* and *Aquicella* at the left, and *L. pneumophila* at the lower right. Genes are indicated by coloured rectangles, and HGT is indicated by the curved arrows. The divergence of *Aquicella* and the non-*pneumophila Legionella* species is indicated with vertical, dashed arrows. *L. yabuuchiae* is an example of a species that shares 7–10 of 25 effectors with *L. pneumophila; L. longbeachae* shares 18/25 effectors; and *L. fallonii* is representative of species sharing 21 or 22 of 25 effectors. Within the infected amoebae hosts in the centre of the figure, the black circles represent the nucleus of the protozoan hosts, whereas the white circles represent contractile vacuoles.

Based on these genomic data, we can also posit a scenario by which the *L. pneumophila* T2SS evolved (Fig. 10). To begin, it is hypothesized that the T2SS emerged within a common ancestor of *Aquicella*, *Berkiella* and *Legionella*, helping to promote an intra-amoebal lifestyle. The T2SS was lost within *Berkiella* species, and perhaps this event had some connection to the *B* *erkiellae* becoming obligate intracellular parasites, targeting the host cell nucleus for survival. The acquisition of core effector genes (e.g. *proA*) probably helped to shape the early evolution of the *Aquicella–Legionella* ancestor (Fig. 10, step 1). With time, *Aquicella* and *Legionella* diverged from each other (Fig. 10, step a), although both retained their T2SS and remained as facultative intracellular parasites of amoebae. The ancestral *Legionella* appears to have acquired additional effectors via inter-kingdom HGT, owing to its natural competence and ability to incorporate environmental DNA. As one example of HGT, *Legionella* probably acquired genetic material from its protozoan host, giving rise to eukaryotic-like T2SS substrates such as LapA (Fig. 10, step 2). We predict that a number of the T2SS substrates that are currently described as ‘novel’ will be re-classified as eukaryotic-like as more protozoan genomes are sequenced. While growing within its amoebal hosts, *Legionella* probably encountered giant viruses (mimiviruses) that also parasitize protozoa. This co-habitation may have provided another conduit for the HGT of effectors, including the T2SS substrate ChiA (Fig. 10, step 3). When *Legionella* emerges from its spent protozoan hosts, it encounters a wide variety of other organisms in its aquatic environment, such as cyanobacteria, water moulds and red algae. This undoubtedly provided yet additional opportunities for gene acquisition, accounting for the T2SS substrates GamA, Lcl and LirB, among others (Fig. 10, step 4). It is reasonable to think that *Legionella*’s host range grew as its T2SS effector repertoire expanded. Consequently, *Legionella* may have shared ecological niches with other intracellular bacterial pathogens, such as *Francisella* species, and thereby acquired further effectors, such as Map, NttG and SrnA, via inter-bacterial HGT (Fig. 10, step 5). Ultimately, the acquisition of even more T2SS effectors, along with other events, such as the evolution of the Dot/Icm T4SS, led to the emergence of the *L. pneumophila* species (Fig. 10, step 6). Based upon the differences in the known-effector repertoire amongst the *Legionella* species (Fig. 8), we posit that each of the different *Legionella* species/clades travelled along their own path of T2SS evolution, which probably includes the acquisition of T2SS substrates that do not have homologues in *L. pneumophila* (Fig. 10, steps b, c and d).

In the coming years, we anticipate the discovery of additional *L. pneumophila*-like T2SSs and new genome sequences that will provide further insight into the diverse origins of the many effectors in the expansive genus *Legionella*. Finally, the genomic analysis of the *L. pneumophila* T2SS that has been reviewed here can serve as a model for the investigation of other bacterial T2SSs, especially those that are present in aquatic and/or intracellular parasites of protozoa.

## Supplementary Data

Supplementary File 1
