# Supplementary material for: Assessing the impact, genomics and evolution of type II secretion across a large, medically important genus: the Legionella type II secretion paradigm
Source: Microb Genom. 2019 Jun 5;5(6):e000273. doi: 10.1099/mgen.0.000273 (PMC6617341; doi:10.1099/mgen.0.000273)
Supplement: Supplementary File 1 [file mgen-5-273-s001.pdf]

Supplemental Table 1: Genera of Gram-negative bacteria lacking a type II secretion system[a]

Acidobacteria
- Acidobacterium
- Chloracidobacterium
- Granulicella
- Koribacter
- Terriglobus
- Solibacter

Aquificae
- Aquifex
- Hydrogenobacter
- Hydrogenobaculum
- Thermocrinis
- Desulfurobacterium
- Thermovibrio
- Persephonella
- Sulfurihydrogenibium

Armatimonadetes
- Chthonomonas

Bacteroidetes
- Bacteroides
- Cardinium
- Odoribacter
- Paludibacter
- Parabacteroides
- Porphyromonas
- Tannerella
- Prevotella
- Alistipes
- Azobacteroides
- Belliella
- Cyclobacterium
- Echinicola
- Cytophaga
- Dyadobacter
- Emticicia
- Fibrella
- Flexibacter
- Leadbetterella
- Runella
- Spirosoma
- Marivirga
- Sulcia
- Blattabacterium
- Fluviicola
- Owenweeksia
- Aequorivita
- Capnocytophaga
- Cellulophaga
- Croceibacter
- Flavobacterium
- Gramella
- Krokinobacter
- Lacinutrix
- Maribacter
- Muricauda
- Nonlabens
- Ornithobacterium
- Polaribacter
- Psychroflexus
- Riemerella
- Robiginitalea
- Weeksella
- Zobellia
- Zunongwangia
- Uzinura
- Chitinophaga
- Niastella
- Haliscomenobacter
- Saprospira
- Pedobacter
- Solitalea
- Sphingobacterium
- Rhodothermus
- Salinibacter
- Amoebophilus

Caldiserica
- Caldisericum

Chlamydiae
- Chlamydophila
- Parachlamydia
- Protochlamydia
- Simkania
- Waddlia

Chlorobi
- Chlorobaculum
- Chlorobium
- Chloroherpeton
- Pelodictyon
- Prosthecochloris
- Ignavibacterium
- Melioribacter

Cyanobacteria
- Gloeobacter
- Anabaena
- Cylindrospermum
- Nostoc
- Calothrix
- Rivularia
- Geitlerinema
- Crinalium
- Trichodesmium
- Pleurocapsa
- Halothece
- Dactylococcopsis
- Cyanobacterium
- Gloeocapsa
- Microcystis
- Synechocystis
- Chroococcidiopsis
- Cyanothece
- Microcoleus
- Oscillatoria
- Stanieria
- Acaryochloris
- Chamaesiphon
- Leptolyngbya
- Prochlorococcus
- Pseudanabaena
- Cyanobium
- Synechococcus
- Thermosynechococcus

Deferribacteres
- Calditerrivibrio
- Deferribacter
- Denitrovibrio
- Flexistipes

Dictyoglomi
- Dictyoglomus

Elusimicrobia
- Elusimicrobium

Fibrobacteres
- Fibrobacter

Fusobacteria
- Fusobacterium
- Ilyobacter
- Leptotrichia
- Sebaldella
- Streptobacillus

Gemmatimonadetes
- Gemmatimonas

Nitrospirae
- Nitrospira
- Leptospirillum
- Thermodesulfovibrio

Planctomycetes
- Phycisphaera
- Isosphaera
- Pirellula
- Planctomyces
- Rhodopirellula
- Singulisphaera

Proteobacteria

-**Alphaproteobacteria**
- Asticcacaulis
- Brevundimonas
- Magnetococcus
- Bartonella
- Beijerinckia
- Methylocella
- Bradyrhizobium
- Nitrobacter
- Oligotropha
- Rhodopseudomonas
- Brucella
- Ochrobactrum
- Hyphomicrobium
- Pelagibacterium
- Rhodomicrobium
- Methylobacterium
- Methylocystis
- Chelativorans
- Mesorhizobium
- Parvibaculum
- Agrobacterium
- Liberibacter
- Rhizobium
- Sinorhizobium
- Azorhizobium
- Starkeya
- Xanthobacter
- Hodgkinia
- Dinoroseobacter
- Jannaschia
- Ketogulonicigenium
- Ketogulonigenium
- Octadecabacter
- Paracoccus
- Phaeobacter
- Pseudovibrio
- Rhodobacter
- Roseobacter
- Ruegeria
- Silicibacter
- Acetobacter
- Acidiphilium
- Gluconacetobacter
- Gluconobacter
- Granulibacter
- Azospirillum
- Endolissoclinum
- Magnetospirillum
- Rhodospirillum
- Tistrella
- Anaplasma
- Ehrlichia
- Neorickettsia
- Wolbachia
- Orientia
- Rickettsia
- Pelagibacter
- Midichloria
- Erythrobacter
- Zymomonas
- Micavibrio
- Polymorphum
- Puniceispirillum

-**Betaproteobacteria**
- Advenella
- Taylorella
- Polynucleobacter
- Herbaspirillum
- Herminiimonas
- Janthinobacterium
- Zinderia

Gallionella
Thiobacillus
Methylobacillus
Methylotenera
Methylovorus
Laribacter
Neisseria
Pseudogulbenkiania
Nitrosomonas
Azoarcus
Dechloromonas
Dechlorosoma
Accumulibacter
Kinetoplastibacterium
Nasuia
Tremblaya

-**Deltaproteobacteria**

Bacteriovorax
Bdellovibrio
Desulfarculus
Desulfatibacillum
Desulfobacterium
Desulfobacula
Desulfococcus
Desulfobulbus
Desulfocapsa
Desulfotalea
Desulfohalobium
Desulfomicrobium
Desulfovibrio
Lawsonia
Hippea
Pelobacter
Anaeromyxobacter
Corallococcus
Myxococcus
Stigmatella
Haliangium
Sorangium
Desulfobacca
Desulfomonile
Syntrophus
Syntrophobacter

-**Epsilonproteobacteria**

Arcobacter
Campylobacter
Sulfurospirillum
Helicobacter
Sulfuricurvum
Sulfurimonas
Wolinella
Hydrogenimonas
Caminibacter
Cetia
Lebetimonas
Nautilia
Nitratiruptor
Sulfurovum
Nitratifractor

-**Gammaproteobacteria**

Acidithiobacillus
Oceanimonas
Dichelobacter
Allochromatium
Nitrosococcus
Thiocystis
Thioflavicoccus
Spiribacter
Halothiobacillus
Blochmannia
Buchnera
Cronobacter
Edwardsiella
Moranella
Morganella
Pantoea
Photorhabdus
Proteus
Providencia
Riesia
Salmonella
Sodalis
Wigglesworthia
Xenorhabdus
Berkiella
Coxiella
Rickettsiella
Methylococcus
Methylomicrobium
Chromohalobacter
Portiera
Marinomonas
Actinobacillus
Aggregatibacter
Bibersteinia
Gallibacterium
Haemophilus
Mannheimia
Pasteurella
Moraxella
Psychrobacter
Azotobacter
Francisella
Cycloclasticus
Methylophaga
Piscirickettsia
Frateuria
Rhodanobacter
Xylella
Baumannia
Ruthia
Carsonella
Vesicomyosocius

Spirochaetes

Brachyspira
Leptospira
Turneriella
Borrelia
Sphaerochaeta
Spirochaeta
Treponema

Synergistetes

Aminobacterium
Anaerobaculum
Thermanaerovibrio
Thermovirga

Tenericutes

Acholeplasma
Phytoplasma
Mesoplasma
Spiroplasma
Mycoplasma
Ureaplasma

Thermodesulfobacteria

Thermodesulfatator
Thermodesulfobacterium

Thermotogae

Fervidobacterium
Kosmotoga
Marinitoga
Mesotoga
Petrotoga
Thermosipho
Thermotoga

Verrucomicrobia

Opitutus
Coraliomargarita
Methylacidiphilum
Akkermansia

[a] The absence of T2SS was determined as previously described (1), using HMMs enabling detection and discrimination of T2SS from the closely related Type IV and Tad pili.

Supplemental Table 2: Putative T2SS substrates of *L. pneumophila* [a]

Strain Phil-1 ORF	Strain 130b ORF	Protein activity or sequence novelty	Location(s)[b]	Role in infection (if known) [c]	Crystal structure	References
lpg0041	*unknown* [d]	Ser-Thr-rich glycosyl-phosphatidyl-inositol-anchored membrane family	Sup't, OMV			*(2-4)*
lpg0042	*lpw00411*	Novel	Sup't, OMV			*(2)*
lpg0085	*lpw00831*	putative ATP-dependent zinc protease	Sup't		PDB ID: 2PMA	*(4)*
lpg0101	*lpw01011*	opacity protein and related surface antigens	Sup't, OMV			*(2, 4)*
lpg0165	*lpw02591*	uncharacterized conserved protein	Sup't, OMV			*(2, 4)*
lpg0187	*lpw02791*	zinc metalloprotein, probable succinyl-diaminopimelate desuccinylase	Sup't, OMV			*(2, 4)*
lpg0198	*unknown* [d]	uncharacterized protein	Sup't			*(4)*
lpg0301	*lpw03931*	eukaryotic-like protein of uncharacterized function (DUF3421)	Sup't			*(4)*
lpg0374	*lpw04571*	Novel	Sup't, OMV			*(2, 4)*
lpg0482	*lpw05621*	endo-1,4 beta-glucanase/peptidase M42 family protein	Sup't, OMV			*(2, 4)*
lpg0497	*lpw05761*	adenosine deaminase	Sup't			*(2)*
lpg0708	*lpw07851*	IcmL-like macrophage killing protein with similarity to conjugation protein	Sup't			*(2)*
lpg0712 (*yjeA*)	*lpw07891*	endo-1,4-beta-xylanase-like protein of uncharacterized function (DUF3298)	Sup't, OMV			*(2, 4)*
lpg0798	*lpw08781*	protein of uncharacterized function (DUF3757)	Sup't, OMV			*(2)*
lpg0804	*lpw08841*	cholylglycine hydrolase (penicillin acylase)	Sup't			*(3)*
lpg0877	*lpw09611*	Putative transporter	Sup't			*(4)*
lpg0957	*lpw10431*	uncharacterized protein, similar to lipase	Sup't, OMV			*(2, 4)*
lpg0971	*lpw10571*	eukaryotic-like ecto-ATP diphosphohydrolase II	Sup't, OMV	promotes growth in Ac, THP-1, and murine lung		*(2, 5)*
lpg1030	*lpw22421*	Uncharacterized protein	Sup't, OMV			*(2, 4)*
lpg1156	*lpw12101*	Bacterial leucyl aminopeptidase precursor	Sup't			*(2, 4)*
lpg1233	*unknown* [d]	Uncharacterized protein	Sup't			*(2, 4)*
lpg1244 (*lvrE*)	*lpw01541*	protein of uncharacterized function (DUF1566) LvrE	Sup't (Tat substrate)			*(3, 6)*
lpg1318	*lpw13271*	uncharacterized protein	Sup't			*(2, 4)*
lpg1431	*lpw14481*	uncharacterized protein	Sup't, OMV			*(2, 4)*
lpg1585	*lpw16101*	EnhB-like	Sup't, OMV			*(4)*

lpg1645	*lpw16711*	uncharacterized conserved protein	Sup't			*(4)*
lpg1647	*lpw16731*	uncharacterized YceI-like domain-containing protein	Sup't			*(4)*
lpg1655	*lpw16811*	metalloprotease precursor, similar to ProA	Sup't			*(2, 4)*
lpg1667	*lpw16931*	IgA peptidase M64	Sup't (T4SS substrate)			*(2-4, 7)*
lpg1910	*lpw19501*	D-alanyl-D-alanine carboxypeptidase precursor	Sup't			*(2, 4)*
lpg1993	*lpw20501*	polysaccharide deacetylase family sporulation protein PdaB	Sup't			*(4)*
lpg2019	*lpw20771*	extracellular serine metalloprotease precursor	Sup't			*(2-4)*
lpg2206 (*wipC*)	*lpw23891*	uncharacterized protein (paralog of T4SS effectors WipA/WipB)	Sup't			*(2, 4, 8)*
lpg2217	*lpw24031*	probable bifunctional chitinase/lysozyme precursor	Sup't			*(2)*
lpg2220	*lpw24061*	Novel	Sup't			*(2-4)*
lpg2222 (*lpnE*)	*lpw24081*	eukaryotic-like putative beta-lactamase hcpC precursor, similar to EnhC	Sup't, OMV (possible T4SS substrate)	promotes entry into THP-1 and A549, but not required for growth in THP-1 and A549	PDB ID: 6DEH	*(2, 9-11)*
lpg2246	*lpw24351*	Novel	Sup't			*(2, 4)*
lpg2275	*lpw24661*	uncharacterized protein	Sup't, OMV	not required for growth in Ap and U937		*(2, 12)*
lpg2320	*lpw25101*	Novel	Sup't (Tat substrate)			*(2-4)*
lpg2397	*lpw26091*	peptidase	Sup't			*(4)*
lpg2443	*lpw26631*	Novel	Sup't (T4SS substrate)			*(4, 7)*
lpg2586	*lpw28341*	papain family cysteine protease	Sup't			*(4)*
lpg2588 (*legS1*)	*lpw28361*	calcineurin-like phosphoesterase superfamily domain	Sup't			*(2, 4)*
lpg2596	*lpw28451*	LysM domain/BON superfamily protein	Sup't, OMV			*(2)*
lpg2607 (*pepO*)	*lpw28561*	neutral metallo-endopeptidase PepO	Sup't, OMV (T4SS substrate)			*(2, 4, 13)*
lpg2677	*lpw29311*	5'-nucleotidase/endonuclease YhcR precursor	Sup't			*(2-4)*
lpg2819 (*lppA*)	*lpw30761*	HopD2-like tyrosine phosphatase II superfamily protein	Sup't	promotes growth in Ac and Dd under phytate-rich conditions	PDB ID: 4TVV	*(4, 14)*

[a] based on the presence of the indicated protein in wild-type culture supernatants, plus the presence of a secretion signal at the N-terminus of the predicted protein

[b] Sup't, protein is present in broth culture supernatant; OMV, also present in outer membrane vesicles; Surface, also present on the bacterial cell surface. Proteins that are predicted to be Tat, rather than Sec, substrates are indicated in parentheses.

[c] based upon the behavior of the corresponding mutant(s) in the indicated infection assay(s). "not required", when the mutant was not different from wildtype; "promotes", when the mutant was impaired relative to wild type, and that defect was reversed by genetic complementation. Ac: *Acanthamoeba castellanii*; Ap: *Acanthamoeba polyphaga*; Wm: *Willaertia magna*; Dd: *Dictyostelium discoideum*

[d] the absence of the corresponding annotated ORF in strain 130b may be due to gaps in the draft genome sequence.

Supplemental Table 3. Overview of T2S effector gene regulation.

T2S Effector Gene	Promoters [a]	Notes on regulation [b]	Expression during various growth phases (relative to E phase in broth) [c]	References
celA (lpw19571)	2	• expression downregulated in PE in *pmrA* and *pmrB* mutants<br>• induced by nicotinic acid and within biofilms	• downregulated in PE phase in BYE<br>• upregulated in NI<br>• no change in Ac and U937	(15-18)
chiA (lpw11641)	1	• expression downregulated in PE in *pmrA* and *pmrB* mutants<br>• highly downregulated in E and PE in *cpxRA* mutant<br>• induced by nicotinic acid, chlorine treatment, and water stress	• upregulated in PE phase in BYE<br>• upregulated in Ac, NI, and U937	(15-17, 19-21)
gamA (lpw05041)	1 (in operon)	• expression downregulated in *csrA* mutant<br>• downregulated in E in *cpxRA* mutant<br>• repressed by nicotinic acid treatment and water stress	• no change in PE phase in BYE<br>• no change in U937<br>• upregulated in Ac and NI	(15, 17, 20-22)
lapA (lpw30701)	2	• expression downregulated in E in *cpxRA* mutant<br>• expression downregulated during water stress<br>• antisense ncRNA (lppnc0666) in coding region	• no change in PE phase in BYE<br>• upregulated in Ac, NI, and U937<br>• expression is further increased in Ac in the absence of PlaC	(15, 20, 21, 23)
lapB (lpw00321)	1 (in operon)	• antisense ncRNA (lppnc0011) in coding region of first gene in operon	• upregulated in PE phase in BYE<br>• upregulated in Ac, NI, and U937<br>• expression is further increased in Ac in the absence of PlaC	(15, 23)
legP (lpw32851)	1	• expression upregulated in PE in *pmrA* mutant<br>• downregulated in E in *cpxRA* mutant<br>• upregulated during water stress	• upregulated in PE phase in BYE<br>• upregulated in Ac, NI, and U937	(15, 16, 20, 21)
lipA (lpw05481)	3	• secreted nonphospholipid lipase activity upregulated in *letA*[d] and *rpoS*[d] mutants<br>• expression upregulated during water stress	• downregulated in PE phase in BYE<br>• no change in Ac, NI, and U937	(15, 20, 24)
lipB (lpw12111)	1	• expression downregulated in *rpoS* mutant<br>• expression downregulated during water stress	• downregulated in PE phase in BYE<br>• no change in Ac<br>• upregulated in NI and THP-1 and U937 cells	(15, 20, 24-26)

lirB (lpw20131)		• expression downregulated in E and PE in *cpxRA* mutant<br>• upregulated during water stress<br>• repressed by nicotinic acid	• downregulated in PE phase in BYE	(17, 20, 21, 27)
lcl (lpw28961)		• expression downregulated in E in *cpxRA* mutant<br>• upregulated in *rpoS* mutant	• downregulated in PE phase in BYE	(21, 26, 27)
lpw03521		• repressed by nicotinic acid	• downregulated in PE phase in BYE	(27)
map (lpw11671)	1	• secreted phosphatase activity slightly decreased in *letA* and *rpoS* mutants<br>• expression downregulated in PE in *pmrA* and *pmrB* mutants<br>• downregulated in E in *csrA* mutant	• downregulated in PE phase in BYE<br>• upregulated in Ac, NI,<br>• no change in U937 cells	(15, 16, 22, 24)
nttA (lpw13951)	1	• expression upregulated in PE in *pmrB* mutant<br>• upregulated in E in *csrA* mutant<br>• downregulated in PE in *cpxRA* mutant<br>• downregulated in *rpoS* mutant<br>• induced by nicotinic acid and water stress	• upregulated in PE phase in BYE<br>• no change in Ac and NI<br>• downregulated in U937 cells	(15-17, 20-22, 26)
nttB (lpw28721)	1	• expression upregulated in E in *csrA* mutant<br>• downregulated in PE in *cpxRA* mutant<br>• downregulated during water stress<br>• antisense ncRNA (lppnc0622) in coding region	• upregulated in PE phase in BYE<br>• upregulated in Ac and NI<br>• downregulated in U937	(15, 20-23)
nttC (lpw18401)	1	• differentially expressed during water treatment (induced early and repressed late)	• no change in PE phase in BYE<br>• no change in Ac<br>• downregulated in NI and U937 cells	(15, 20)
nttD (lpw10421)	1 (in operon)	• expression downregulated in E and PE in *cpxRA* mutant<br>• repressed by nicotinic acid treatment and water stress<br>• antisense ncRNA (lppnc0243) in coding region of first gene in operon	• downregulated in PE phase in BYE<br>• no change in Ac<br>• downregulated in NI and U937 cells	(15, 17, 20, 21, 23)
nttE (lpw02811)		• expression downregulated in E in *cpxRA* mutant	• upregulated in PE phase in BYE<br>• upregulated in THP-1 cells	(21, 25, 27)
nttF (lpw09571)		• expression induced by nicotinic acid<br>• downregulated in E in *csrA* mutant	• upregulated in PE phase in BYE	(17, 22, 27)
nttG (lpw18641)			• downregulated in PE phase in BYE<br>• upregulated in THP-1cells	(25, 27)

plaA (lpw25361)	1	• secreted lysophospholipase A activity decreased in *letA*[d] and *rpoS*[d] mutants<br>• expression downregulated during water stress	• downregulated in PE phase in BYE<br>• no change in Ac, NI, and U937 cells	(15, 20, 24)
plaC (lpw30971)	1	• expression and secreted GCAT activity decreased in *letA* and *rpoS* mutants<br>• expression upregulated in E in *csrA* mutant<br>• downregulated in PE in *cpxRA* mutant<br>• downregulated during water stress	• upregulated in PE phase in BYE<br>• upregulated in Ac, NI, and U937 cells<br>• expression is further increased in Ac in the absence of LapA and LapB	(15, 20-22, 24)
plcA (lpw05821)	1	• secreted p-NPPC hydrolase activity decreased in *letA*[d] and *rpoS*[d] mutants	• no change in PE phase in BYE<br>• no change in Ac, NI, and U937 cells	(15)
plcB (lpw14741)	1	• expression downregulated in *rpoS* mutant and secreted p-NPPC hydrolase activity decreased in *letA*[d] and *rpoS* mutants<br>• induced by nicotinic acid, chlorine treatment, and water stress	• upregulated in PE phase in BYE<br>• upregulated in Ac and THP-1<br>• no change in NI and U937 cells	(15, 17, 19, 20, 25, 26)
proA (lpw05471)	1	• secreted protease activity decreased in *letA*[d] and *rpoS*[d] mutants<br>• downregulated in E in *cpxRA* mutant<br>• downregulated during water stress	• upregulated in PE phase in BYE<br>• upregulated in Ac and NI<br>• no change in U937 cells	(15, 20, 21, 24)
srnA (lpw31111)	1	• secreted RNAse activity not affected in *letA*[d] and *rpoS*[d] mutants<br>• expression downregulated in E in *cpxRA* mutant<br>• expression downregulated during water stress	• no change in PE phase in BYE<br>• upregulated in Ac, NI, THP-1, and U937 cells	(15, 20, 21, 24)

[a] Presence of promoters and ncRNA were based on transcriptional deep sequencing of *L. pneumophila* strain Paris (23)

[b] ncRNA: non-coding RNA; E: exponential-phase; PE: post-exponential phase

[c] Ac: *Acanthamoeba castellanii*; NI: *Naegleria lovaniensis*

[d] Gene expression not monitored in the indicated mutant *L. pneumophila* strain

References

1. Abby SS, Cury J, Guglielmini J, Neron B, Touchon M, Rocha EP. Identification of protein secretion systems in bacterial genomes. Sci Rep. 2016;6:23080.
2. Galka F, Wai SN, Kusch H, Engelmann S, Hecker M, Schmeck B, et al. Proteomic characterization of the whole secretome of *Legionella pneumophila* and functional analysis of outer membrane vesicles. Infect Immun. 2008;76(5):1825-36.
3. De Buck E, Hoper D, Lammertyn E, Hecker M, Anne J. Differential 2-D protein gel electrophoresis analysis of *Legionella pneumophila* wild type and Tat secretion mutants. Int J Med Microbiol. 2008;298(5-6):449-61.
4. Aurass P, Gerlach T, Becher D, Voigt B, Karste S, Bernhardt J, et al. Life Stage-specific Proteomes of *Legionella pneumophila* Reveal a Highly Differential Abundance of Virulence-associated Dot/Icm effectors. Mol Cell Proteomics. 2016;15(1):177-200.
5. Riedmaier P, Sansom FM, Sofian T, Beddoe T, Schuelein R, Newton HJ, et al. Multiple ecto-nucleoside triphosphate diphosphohydrolases facilitate intracellular replication of *Legionella pneumophila*. Biochem J. 2014;462(2):279-89.
6. DebRoy S, Dao J, Soderberg M, Rossier O, Cianciotto NP. *Legionella pneumophila* type II secretome reveals unique exoproteins and a chitinase that promotes bacterial persistence in the lung. Proceedings of the National Academy of Sciences of the United States of America. 2006;103(50):19146-51.
7. Zhu WH, Banga S, Tan YH, Zheng C, Stephenson R, Gately J, et al. Comprehensive Identification of Protein Substrates of the Dot/Icm Type IV Transporter of *Legionella pneumophila*. Plos One. 2011;6(3).
8. Ninio S, Zuckman-Cholon DM, Cambronne ED, Roy CR. The *Legionella* IcmS-IcmW protein complex is important for Dot/Icm-mediated protein translocation. Mol Microbiol. 2005;55(3):912-26.
9. Newton HJ, Sansom FM, Bennett-Wood V, Hartland EL. Identification of *Legionella pneumophila*-specific genes by genomic subtractive hybridization with *Legionella micdadei* and identification of lpnE, a gene required for efficient host cell entry. Infect Immun. 2006;74(3):1683-91.
10. Voth KA, Chung IYW, van Straaten K, Li L, Boniecki MT, Cygler M. The structure of *Legionella* effector protein LpnE provides insights into its interaction with Oculocerebrorenal syndrome of Lowe (OCRL) protein. FEBS J. 2018.
11. Weber SS, Ragaz C, Hilbi H. The inositol polyphosphate 5-phosphatase OCRL1 restricts intracellular growth of *Legionella*, localizes to the replicative vacuole and binds to the bacterial effector LpnE. Cell Microbiol. 2009;11(3):442-60.
12. Hayashi T, Nakamichi M, Naitou H, Ohashi N, Imai Y, Miyake M. Proteomic Analysis of Growth Phase-Dependent Expression of *Legionella pneumophila* Proteins Which Involves Regulation of Bacterial Virulence Traits. Plos One. 2010;5(7).
13. Price CT, Jones SC, Amundson KE, Kwaik YA. Host-mediated post-translational prenylation of novel dot/icm-translocated effectors of *Legionella pneumophila*. Front Microbiol. 2010;1:131.
14. Weber S, Stirnimann CU, Wieser M, Frey D, Meier R, Engelhardt S, et al. A type IV translocated *Legionella* cysteine phytase counteracts intracellular growth restriction by phytate. The Journal of biological chemistry. 2014;289(49):34175-88.

15. White RC, Gunderson FF, Tyson JY, Richardson KH, Portlock TJ, Garnett JA, et al. Type II Secretion-Dependent Aminopeptidase LapA and Acyltransferase PlaC Are Redundant for Nutrient Acquisition during *Legionella pneumophila* Intracellular Infection of Amoebas. Mbio. 2018;9(2).
16. Al-Khodor S, Kalachikov S, Morozova I, Price CT, Abu Kwaik Y. The PmrA/PmrB two-component system of *Legionella pneumophila* is a global regulator required for intracellular replication within macrophages and protozoa. Infect Immun. 2009;77(1):374-86.
17. Edwards RL, Bryan A, Jules M, Harada K, Buchrieser C, Swanson MS. Nicotinic acid modulates *Legionella pneumophila* gene expression and induces virulence traits. Infect Immun. 2013;81(3):945-55.
18. Hindre T, Bruggemann H, Buchrieser C, Hechard Y. Transcriptional profiling of *Legionella pneumophila* biofilm cells and the influence of iron on biofilm formation. Microbiology. 2008;154(Pt 1):30-41.
19. Bodet C, Sahr T, Dupuy M, Buchrieser C, Hechard Y. *Legionella pneumophila* transcriptional response to chlorine treatment. Water research. 2012;46(3):808-16.
20. Li L, Mendis N, Trigui H, Faucher SP. Transcriptomic changes of *Legionella pneumophila* in water. BMC genomics. 2015;16:637.
21. Tanner JR, Li L, Faucher SP, Brassinga AK. The CpxRA two-component system contributes to *Legionella pneumophila* virulence. Mol Microbiol. 2016;100(6):1017-38.
22. Sahr T, Rusniok C, Impens F, Oliva G, Sismeiro O, Coppee JY, et al. The *Legionella pneumophila* genome evolved to accommodate multiple regulatory mechanisms controlled by the CsrA-system. 2017;13(2):e1006629.
23. Sahr T, Rusniok C, Dervins-Ravault D, Sismeiro O, Coppee JY, Buchrieser C. Deep sequencing defines the transcriptional map of *L. pneumophila* and identifies growth phase-dependent regulated ncRNAs implicated in virulence. RNA biology. 2012;9(4):503-19.
24. Broich M, Rydzewski K, McNealy TL, Marre R, Flieger A. The global regulatory proteins LetA and RpoS control phospholipase A, lysophospholipase A, acyltransferase, and other hydrolytic activities of *Legionella pneumophila* JR32. J Bacteriol. 2006;188(4):1218-26.
25. Faucher SP, Mueller CA, Shuman HA. *Legionella pneumophila* transcriptome during intracellular multiplication in human macrophages. Front Microbiol. 2011;2:60.
26. Hovel-Miner G, Pampou S, Faucher SP, Clarke M, Morozova I, Morozov P, et al. SigmaS controls multiple pathways associated with intracellular multiplication of *Legionella pneumophila*. J Bacteriol. 2009;191(8):2461-73.
27. Weissenmayer BA, Prendergast JG, Lohan AJ, Loftus BJ. Sequencing illustrates the transcriptional response of *Legionella pneumophila* during infection and identifies seventy novel small non-coding RNAs. Plos One. 2011;6(3):e17570.
